# Neural Quantum States Based on Selected Configurations

**DOI:** 10.1021/acs.jpclett.6c00520

**Published:** 2026-04-23

**Authors:** Marco Julian Solanki, Lexin Ding, Markus Reiher

**Affiliations:** Department of Chemistry and Applied Biosciences, 31064 ETH Zürich, Vladimir-Prelog-Weg 2, CH-8093 Zürich, Switzerland

## Abstract

Neural quantum states (NQS) provide a flexible and highly expressive parametrization of wave functions for strongly correlated problems in quantum chemistry. Despite rapid advances in network architectures, the evaluation of electronic energies remains almost exclusively based on variational Monte Carlo (VMC). While VMC is effective for structured systems such as spin chains, its accuracy and efficiency for electronic Hamiltonians are hindered by sharply peaked distributions, stochastic gradient noise, and slow convergence with sample size. In this Letter, we assess the capability of NQS-VMC to efficiently capture correlation in electronic ground states by comparing it to a recently developed NQS-based selected configuration (NQS-SC) approach. We set up a systematic comparison of the ground-state optimizations obtained with NQS-VMC and NQS-SC for molecular systems dominated by either static or dynamical correlation. The comparison demonstrates a clear advantage of NQS-SC over NQS-VMC in both energy accuracy and wave function coefficients, particularly for statically correlated molecules. Moreover, NQS-SC exhibits robust systematic improvability, whereas NQS-VMC does not. These findings position NQS-SC as the new default approach over NQS-VMC for electronic structure calculations. We further observe that neither NQS-SC nor NQS-VMC can efficiently capture dynamical correlation, highlighting the need for future hybrid methods, such as multiconfigurational perturbation theories built on top of NQS solutions.

## Graphical abstract

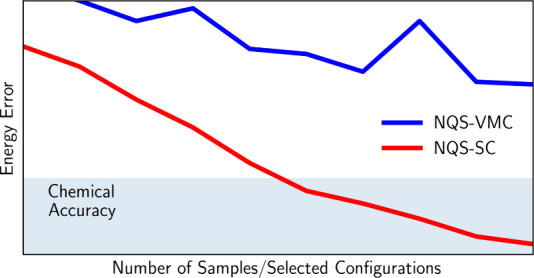

Neural quantum states (NQS) have been proposed as a novel ansatz for solving the many-body Schrödinger equation.^1^ Leveraging the expressive power of artificial neural networks allows NQS to deliver a compact representation for strongly correlated wave functions. Theoretical analyses and numerical studies on lattice Hamiltonians have demonstrated that NQS can represent highly entangled states using a number of network parameters that scales only polynomially with system size.^2,3^ These results on the representational power of NQS have motivated a rapidly growing body of literature exploring increasingly sophisticated network architectures ranging from restricted Boltzmann machines to autoregressive models,^4–16^ some of which have been applied to *ab initio* quantum chemistry.^17–28^ However, the practical scope of NQS for molecular systems remains limited to about 50 electrons in 40 spatial orbitals,^22^ as a central challenge is the efficient access to the exponentially many wave function coefficients required for accurate energy evaluation and optimization.

Currently, the predominant method for evaluating the energy of an NQS is variational Monte Carlo (VMC) sampling. For early NQS, the sampling method of choice was Metropolis–Hastings sampling,^1,19^ while many later NQS implementations applied autoregressive sampling.^21,22,25–27^ The motivation behind this shift was the observation that molecular wave functions are typically dominated by a small subset of configurations (with a long tail of configurations with small weights), leading to high rejection rates during Metropolis–Hastings sampling. For example, ref 19 reported acceptance rates as low as 0.1% for calculations on H_2_O in a 6-31G basis set. The efficiency of Metropolis–Hastings sampling is further limited (i) by the need to prune intermediate samples to obtain approximately independent and identically distributed samples and (ii) by the need for a burn-in period for the sampled Markov chains to equilibrate.^29^ Conceptually, these issues can be resolved with autoregressive sampling, whereby the neural network outputs conditional probabilities that, when sequentially sampled, yield samples from the full probability distribution.^21^ This results in a rejection-free sampling procedure that produces a sequence of uncorrelated samples.

Yet, autoregressive sampling also has its shortcomings. First, it requires specific network architectures, which include an amplitude network to model the probability amplitude of each configuration (such as a recurrent neural network or decoder-only transformer) and a separate phase network for predicting the complex phase of the coefficients.^21,22,25–27^ Second, autoregressive models are unable to directly enforce crucial symmetries in electronic systems, such as fixed particle numbers and magnetizations, thereby necessitating the ad hoc masking/discarding of unphysical configurations.^21,22,25–27^ Finally, the ordering of the orbitals will affect the ground-truth conditional probability distributions, which, in turn, may affect the model’s ability to learn them. Reference 22, for example, used an entanglement-localizing ordering to improve the performance of their model. However, this means that autoregressive models, unlike fully connected NQS, cannot claim invariance to orbital orderings as a potential advantage over competing tensor network methods.

To tackle these issues of VMC for electronic ground states, Li et al. proposed an NQS-based selected configuration (NQS-SC) approach for energy evaluations.^30^ This approach takes strong inspiration from traditional selected configuration interaction (SCI) methods (see, e.g., refs 31–37 ), which have previously been applied to challenging molecular systems governed by both strong correlation^34,38–40^ and system size.^39^ These traditional SCI methods typically select configurations from outside the selected variational space based on information beyond the ansatz for the truncated wave function, such as perturbation theories or criteria that closely resemble them.^34,38,40,41^ NQS-SC, by contrast, selects configurations based on the probability amplitudes predicted by the NQS ansatz, which is defined not only on the selected variational space but also on the external space in which new configurations can arise. The predicted wave function coefficients of these external configurations enter the SC energy implicitly via the so-called local energy.

It has been shown that the nonstochastic nature of NQS-SC can lead to more stable optimizations and higher accuracy in the electronic ground-state energy than NQS-VMC.^24,30,42^ However, as a truncated method, NQS-SC can recover only static correlation. Namely, it can only account for a small fraction of configurations from the exponentially large Hilbert space that make significant contributions to the exact solution. Moreover, the current formulation of the NQS-SC energy as a truncated sum of local energies is not variational, which, if left unchecked, could lead to an underestimation of the energy error.^30^

Hence, a rigorous inspection of NQS-SC relative to NQS-VMC is lacking, but needed to unlock the potential of the NQS parametrization. Therefore, we here evaluate (final) NQS-SC energies variationally in two different ways to facilitate a fair comparison with NQS-VMC. As a result, we can clearly demonstrate the general advantage of NQS-SC over NQS-VMC, which supports the role of SC as a new standard component in NQS parametrizations of electronic ground states.

We start by considering the Hilbert space H of *N* electrons (*N*_α_ spin-up and *N*_β_ spin-down) in 2*L* spin–orbitals (*L* spin-up and *L* spin-down). The second-quantized electronic Hamiltonian can be expressed as (with nucleus-repulsion terms omitted for simplicity)

$$Ĥ=\overset{2L}{\underset{i,j=1}{\sum }}h_{ij}{â}_{i}^{†}{â}_{j}+\frac{1}{2}\overset{2L}{\underset{i,j,k,l=1}{\sum }}V_{ijkl}{â}_{i}^{†}{â}_{j}^{†}{â}_{l}{â}_{k}$$ 1

where *â*_*i*_^(†)^ are fermionic annihilation (creation) operators that annihilate (create) an electron in the *i*-th spin–orbital and *h*_*ij*_ and *V*_*ijkl*_ are one- and two-electron integrals, defined as

$$h_{ij}=-\int \phi_{i}{\left(x\right)}^{*}\left(\frac{1}{2}\nabla_{r}^{2}+\overset{M}{\underset{I=1}{\sum }}\frac{Z_{I}}{\left|r-R_{I}\right|}\right)\phi_{j}\left(x\right)\;dx$$ 2

$$V_{ijkl}=\int \int \phi_{i}{\left(x_{1}\right)}^{*}\phi_{j}{\left(x_{2}\right)}^{*}\frac{1}{\left|r_{1}-r_{2}\right|}\phi_{k}\left(x_{1}\right)\phi_{l}\left(x_{2}\right)\;dx_{1}\;dx_{2}$$ 3

where ϕ_*i*_(***x***) is the *i*-th spin–orbital parametrized by the combined spatial and spin coordinates ***x*** = (***r***, σ) and *I* indexes the system’s *M* nuclei with charges *Z*_*I*_ and coordinates ***R***_*I*_. The exact ground state wave function in such a basis is obtained by variationally minimizing the energy *E* ^FCI^ associated with the full configuration interaction (FCI) ansatz

$$\left|\Psi^{\mathrm{FCI}}\right\rangle =\underset{\left|n\right\rangle \in B}{\sum }\Psi \left(n\right)\left|n\right\rangle$$ 4

where | ***n*** ⟩ = |*n*_1_, *n*_2_, ···, *n*_2*L*_⟩ are occupation number vectors (ONVs) (with *n*_*i*_ = 0, 1 representing the occupation of the *i*-th spin–orbital), which jointly form a complete basis B of H .

The total dimension of the Hilbert space H is determined combinatorically as dim(H)=(LNα)(LNβ) , which steeply constrains the largest numbers of electrons and orbitals that one can describe with FCI in practice, requiring approximations for all but the smallest of systems. We therefore consider an NQS Ψ_**θ**_(***n***) to denote the neural network approximant to the mapping ***n*** 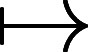 Ψ­(***n***) for a neural network with trainable parameters **θ**.

In the NQS-VMC framework, *n*_sample_ (nonunique) configurations are sampled to approximate the probability distribution induced by the neural network

$$P_{\theta }\left(n\right)=\frac{|\Psi_{\theta }\left(n\right){|}^{2}}{\underset{\left|m\right\rangle \in B}{\sum }|\Psi_{\theta }\left(m\right){|}^{2}}$$ 5

There are many methods to generate samples, with the most widely employed options being (i) the Metropolis–Hastings algorithm, which proposes transitions between configurations and accepts or rejects them based on energetic and probabilistic considerations, and (ii) the autoregressive approach, which decomposes the full probability distribution *P*_**θ**_(***n***) into a sequence of univariate conditional probabilities. The performance of specific samplers can be sensitive to hyperparameter choices, such as the proposal distribution and number of Markov chains for Metropolis–Hastings and the orbital ordering for autoregressive sampling. Moreover, orbital localization can drastically alter the profile of wave function coefficients, thereby affecting the rejection rate and sampling accuracy of the Metropolis–Hastings algorithm. To conclusively evaluate the performance of NQS-VMC without any biases from specific choices of sampler or orbital basis, we therefore chose the most accurate, albeit computationally most expensive, exact Monte Carlo (EMC) sampling method.

In EMC, ONV samples are drawn directly from the distribution *P*_**θ**_(***n***), thereby eliminating all sources of error beyond statistical sampling noise. Once the multiset of samples $S_{\mathrm{MC}}$ has been generated, the energy of the NQS is approximated as

$$E_{\theta }=\underset{\left|n\right\rangle \in B}{\sum }P_{\theta }\left(n\right)E_{\theta }^{\mathrm{loc}}\left(n\right)\approx \frac{1}{\left|S_{\mathrm{MC}}\right|}\underset{\left|n\right\rangle \in S_{\mathrm{MC}}}{\sum }E_{\theta }^{\mathrm{loc}}\left(n\right)\equiv E_{\theta }^{\mathrm{MC}}$$ 6

where $\left|S_{\mathrm{MC}}\right|$ is the number of samples and *E*_**θ**_^loc^(***n***) is the local energy defined as

$$E_{\theta }^{\mathrm{loc}}\left(n\right)=\underset{\left|m\right\rangle \in B}{\sum }\left\langle n\right|Ĥ\left|m\right\rangle \frac{\Psi_{\theta }\left(m\right)}{\Psi_{\theta }\left(n\right)}$$ 7

In EMC, the most deciding factor for accuracy is the number of samples *n*_sample_. According to the central limit theorem, the standard error *δE* of the energy scales as $1/\sqrt{n_{\mathrm{sample}}}$ .^29^ Elementary probability theory also dictates that a configuration | ***n*** ⟩ with probability amplitude *P*_**θ**_(***n***) can only be expected to be accessed if *n*_sample_ is of the order of at least *P*_**θ**_(***n***)^−1^. As the probability distributions of electronic ground states are often dominated by only a small number of important configurations (static correlation), they are, by construction, followed by a long tail of configurations with vanishing weights but considerable collective contribution to the ground-state energy (dynamical correlation). VMC calculations, therefore, often require a vast number of samples to achieve an energy accuracy that can support chemical accuracy of about 1 kcal/mol.^21,26,27^

By contrast, in the SC framework, the weighted sum in eq 6 is truncated to a set of *n*_select_ configurations $S_{\mathrm{select}}$ , which contains the configurations with the highest probability amplitudes predicted by the neural network. After the network parameters are updated, new configurations are selected from the extended set $S_{\mathrm{extend}}=ĤS_{\mathrm{select}}$ consisting of configurations connected to $S_{\mathrm{select}}$ by the electronic Hamiltonian. In practice, we construct $S_{\mathrm{expand}}$ as a random subset of $S_{\mathrm{extend}}$ of size *n*_expand_ and *n*_select_ new configurations are selected from the union $S_{\mathrm{select}}\cup S_{\mathrm{expand}}$ to form the new $S_{\mathrm{select}}$ . The size of $S_{\mathrm{select}}$ is thereby maintained between iterations. The initial $S_{\mathrm{select}}$ consists of the Hartree–Fock configuration as a seed, and all configurations connected to it (or a random subset of them, if the resulting size of the space would otherwise exceed *n*_select_). Motivating this choice is the observation that low-excitation-rank CI wave functions can be a good initial guess for many molecular systems if the variationally optimized Hartree–Fock determinant is taken as a reference for the excitations. We note that this procedure does not rely on the single-reference character of the ground state. Although the Hartree–Fock configuration is used as an initial seed for convenience, the iterative expansion of the selected subspace rapidly incorporates configurations from the full Hilbert space, making the approach equally applicable to multireference systems.

Given a set $S_{\mathrm{select}}$ and some $\left|n\right\rangle \in S_{\mathrm{select}}$ , *P*_**θ**_(***n***) can be approximated as

$$P_{\theta }^{\mathrm{SC}}\left(n\right)=\frac{|\Psi_{\theta }\left(n\right){|}^{2}}{\underset{\left|m\right\rangle \in S_{\mathrm{select}}}{\sum }|\Psi_{\theta }\left(m\right){|}^{2}}$$ 8

The electronic energy can then be estimated as

$$E_{\theta }\approx \underset{\left|n\right\rangle \in S_{\mathrm{select}}}{\sum }P_{\theta }^{\mathrm{SC}}\left(n\right)E_{\theta }^{\mathrm{loc}}\left(n\right)\equiv E_{\theta }^{\mathrm{SC}}$$ 9

As already noted by ref 30, and in contrast to the EMC energy estimate in eq 6, this energy estimate is nonvariational. In our calculations, we recover variationality in two ways. First, the symmetric evaluation of the electronic energy is calculated as

$$E_{\theta }\approx \frac{\underset{\left|m\right\rangle ,\left|n\right\rangle \in S_{\mathrm{select}}}{\sum }{\Psi_{\theta }\left(m\right)}^{*}\left\langle m\right|Ĥ\left|n\right\rangle \Psi_{\theta }\left(n\right)}{\underset{\left|n\right\rangle \in S_{\mathrm{select}}}{\sum }|\Psi_{\theta }\left(n\right){|}^{2}}\equiv E_{\theta }^{\mathrm{SC‐SYM}}$$ 10

This amounts to an energy evaluation with respect to the NQS |Ψ_**θ**_⟩ truncated on $S_{\mathrm{select}}$ . The variationality of this estimate follows from the variational principle for admissible trial wave functions. Second, the variationally optimal energy on $S_{\mathrm{select}}$ is calculated through exact diagonalization. This can be viewed as the result of selected configuration interaction (SCI)

$$E^{\mathrm{SCI}}=\min_{\left|\Phi \right\rangle \in \mathrm{span}\left(S_{\mathrm{select}}\right)}\frac{\left\langle \Phi \right|Ĥ\left|\Phi \right\rangle }{\left\langle \Phi \right|\Phi \rangle }$$ 11

For the purpose of training, *E*_**θ**_^SC^ is still the more suitable objective function, because it is computationally inexpensive and the wave function coefficients of the configurations not only in $S_{\mathrm{select}}$ but also $S_{\mathrm{extend}}$ implicitly enter it through the local energies. The discrepancy between *E*_**θ**_^SC^ and *E*_**θ**_^SC‑SYM^ should be reduced as more and more configurations are selected and finally agree in the limit of $n_{\mathrm{select}}=\dim\left(H\right)$ .

Besides *E*_**θ**_^SC^, the wave function coefficients of the configurations in $S_{\mathrm{extend}}$ also enter the selection process of the new $S_{\mathrm{select}}$ , which favors small wave function values outside of $S_{\mathrm{select}}$ . As such, although the network is formally trained on the selected subspace, the wave function coefficients of configurations outside the selected subspace are implicitly also optimized.

In terms of the computational cost per iteration, NQS-SC and NQS-VMC require the same number of operations for evaluating local energies (which includes finding the connected configurations and evaluating their wave function coefficients) and evaluating the wave function coefficients for the selected/sampled configurations themselves. Additionally, for NQS-SC, the configurations in the union $S_{\mathrm{select}}\cup S_{\mathrm{expand}}$ are sorted and selected according to their wave function amplitudes, leading to an additional overhead in $O\left(n_{\mathrm{select}}\log\left(n_{\mathrm{select}}\right)\right)$ (assuming *n*_expand_ is chosen proportional to *n*_select_). Moreover, we emphasize that, if employed, the exact diagonalization of the selected subspace is performed only once, at the end of the NQS-SC optimization. Therefore, the computational cost of optimizing NQS-SC is not dominated by the exact diagonalization of a Hamiltonian matrix of size *n*_select_ × *n*_select_.

Another crucial factor that determines the performance of a particular NQS implementation is its network architecture. For this, we chose the neural backflow (NBF) architecture first introduced in ref 6 as a variational approximation to the Fermi–Hubbard model. This architecture was later applied to *ab initio* quantum chemistry in ref 24. Recently, it has also been considered in combination with a Jastrow factor^42^ and through the replacement of its feedforward layers with an encoder-only transformer.^28^ It has furthermore been shown to be capable of learning wave functions of highly entangled fermionic systems whose entanglement scales with the system volume, with only a polynomial scaling in terms of network parameters. Such an entanglement structure cannot be efficiently captured by locally connected parametrizations such as matrix product states or finite-order coupled cluster theories, where higher-order excitations are exponentially suppressed. Beyond fermions, NBF has also been adapted for bosons and applied to anharmonic vibrational structure calculations.^43^

We briefly review the NBF model. We start by considering a single Slater determinant (SD) state

$$\left|\Psi^{\mathrm{SD}}\right\rangle ={\mathrm{f̂}}_{1}^{†}{\mathrm{f̂}}_{2}^{†}...{\mathrm{f̂}}_{N}^{†}\left|0\right\rangle$$ 12

where *f̂ *_*j*_^†^ = ∑_*i*=1_^2*L*^ *C*_*ij*_ *â*_*i*_^†^ creates an electron in the orbital specified by the *j*-th column of the orbital coefficient matrix ***C***. |Ψ^SD^⟩ can be expanded in the basis of ONVs of the form | ***n*** ⟩ = ∏_*i*=1_^2*L*^(*â*_*i*_^†^)^*n*_*i*_^|0⟩ as

$$\begin{array}{rll} \left|\Psi^{\mathrm{SD}}\right\rangle & =\left(\overset{2L}{\underset{i_{1}}{\sum }}C_{i_{1}1}{â}_{i_{1}}^{†}\right)\left(\overset{2L}{\underset{i_{2}}{\sum }}C_{i_{2}2}{â}_{i_{2}}^{†}\right)...\left(\overset{2L}{\underset{i_{N}}{\sum }}C_{i_{N}N}{â}_{i_{N}}^{†}\right)\left|0\right\rangle & \\ & =\overset{2L}{\underset{i_{1}i_{2}\cdots i_{N}}{\sum }}C_{i_{1}1}C_{i_{2}2}...C_{i_{N}N}{â}_{i_{1}}^{†}{â}_{i_{2}}^{†}...{â}_{i_{N}}^{†}\left|0\right\rangle \\ & =\overset{2L}{\underset{i_{1}<i_{2}<\cdots <i_{N}}{\sum }}\left(\underset{\pi \in S_{N}}{\sum }\mathrm{sgn}\left(\pi \right)C_{i_{\pi \left(1\right)}1}C_{i_{\pi \left(2\right)}2}\cdots C_{i_{\pi \left(N\right)}N}\right){â}_{i_{1}}^{†}{â}_{i_{2}}^{†}...{â}_{i_{N}}^{†}\left|0\right\rangle , \end{array}$$ 13

where π are elements of the permutation group *S*_*N*_ and sgn­(π) ∈ { ± 1} is the sign of the permutation. In other words, the expansion coefficient Ψ^SD^(***n***) can be expressed as the determinant

$$\Psi^{\mathrm{SD}}\left(n\right)=\left\langle n\right|\Psi^{\mathrm{SD}}\rangle =\det C\left(n\right)$$ 14

where ***C*** (***n***) is a square matrix consisting of rows of the orbital coefficient matrix ***C*** whose indices *i* satisfy *n*_*i*_ = 1.

Equation 14 reveals the structure of the expansion coefficients Ψ^SD^(***n***) of the SD state. Namely, they are coordinated through the determinants of different submatrices of the *same* orbital coefficient matrix ***C***. An NQS directly learning the Ψ^SD^(***n***) faces the nontrivial task of learning this determinant structure, even though |Ψ^SD^⟩ is a simple mean field state without any electron correlation.

The NBF model makes use of this insight and expands it by learning, for each ONV ***n***, a *different* orbital coefficient matrix ***C*** ^(***n***)^. The coefficient Ψ^NBF^(***n***) is then given by the backflow (i.e., ONV-dependent) determinant

$$\Psi^{\mathrm{NBF}}\left(n\right)=\det C^{\left(n\right)}\left(n\right)$$ 15

where ***C*** ^(***n***)^(***n***) is again understood to consist of rows of ***C*** ^(***n***)^ whose indices *i* satisfy *n*_*i*_ = 1. This way, the task of learning the determinant structure is offloaded from the network. At the same time, Ψ^NBF^(***n***) can, thanks to the ONV-dependence of ***C*** ^(***n***)^ and in contrast to Ψ^SD^(***n***) in eq 14, vary freely and therefore introduce electron correlation.

In practice, NBF can be set to learn more than one orbital coefficient matrix for each ONV. An ONV ***n*** is passed through *K* ≥ 1 feedforward layers with widths *d*_*k*_, each performing a linear transformation ***y*** ^(*k*)^ = ***W*** ^(*k*)^ ***x*** ^(*k*–1)^ + ***b*** ^(*k*)^ and a nonlinear activation ***x*** ^(*k*)^ = **σ**(***y*** ^(*k*)^). The weights $W^{\left(k\right)}\in R^{d_{k}\times d_{k-1}}$ and biases $b^{\left(k\right)}\in R^{d_{k}}$ are network parameters to be optimized. The final (activation-less) layer’s output ***y*** ^(*K*)^ has length *d*_*K*_ = *D* × 2*L* × *N* and is reshaped into *D* matrices with dimensions 2*L* × *N* denoted as $C_{1}^{\left(n\right)},C_{2}^{\left(n\right)},...,C_{D}^{\left(n\right)}\in R^{2L\times N}$ . Using these *D* matrices, the coefficient Ψ_**θ**_^NBF^(***n***) predicted by the NBF network for the ONV ***n*** is given by

$$\Psi_{\theta }^{\mathrm{NBF}}\left(n\right)=\overset{D}{\underset{i=1}{\sum }}\det C_{i}^{\left(n\right)}\left(n\right)$$ 16

where **θ** collects the weights ***W*** ^(*k*)^ and biases ***b*** ^(*k*)^.

The cost of training and evaluating the NBF model for a molecular system with *N* electrons in *L* spatial orbitals will scale roughly as $O\left(N^{3}+\mathrm{LN}\right)$ . The $O\left(N^{3}\right)$ contribution comes from the backflow determinant calculations, which are implemented through dense matrix factorizations, while the O(LN) contribution comes from the final feedforward layer having a width of *D* × 2*L* × *N*.

In the following, the NBF model trained with EMC sampling and selected configurations will be referred to as NBF-VMC and NBF-SC, respectively. Neural networks were trained using NetKet,^44,45^ JAX,^46^ and Flax.^47^ Hartree–Fock, MP2, and FCI calculations were performed with PySCF.^48–50^ Variational energy evaluations according to eqs 10 and 11 were performed with PyCI.^51^ The molecular structures employed (apart from the stretched N_2_ molecule and the stretched H_8_ chain) are based on published experimental measurements and were retrieved from ref 52. All energies are measured in Hartree (Ha).

## Stretched N_2_

We start our comparison with a prototypical example with static correlation, namely the stretched N_2_ molecule (with a bond length of 2.25 Å). To limit our memory consumption during training and retain our ability to compute an FCI reference solution, we chose a minimal STO-3G basis set. We present the probability amplitudes predicted and energy errors incurred by NBF-VMC and NBF-SC in Figures 1 and 2, respectively.

**Figure 1:**
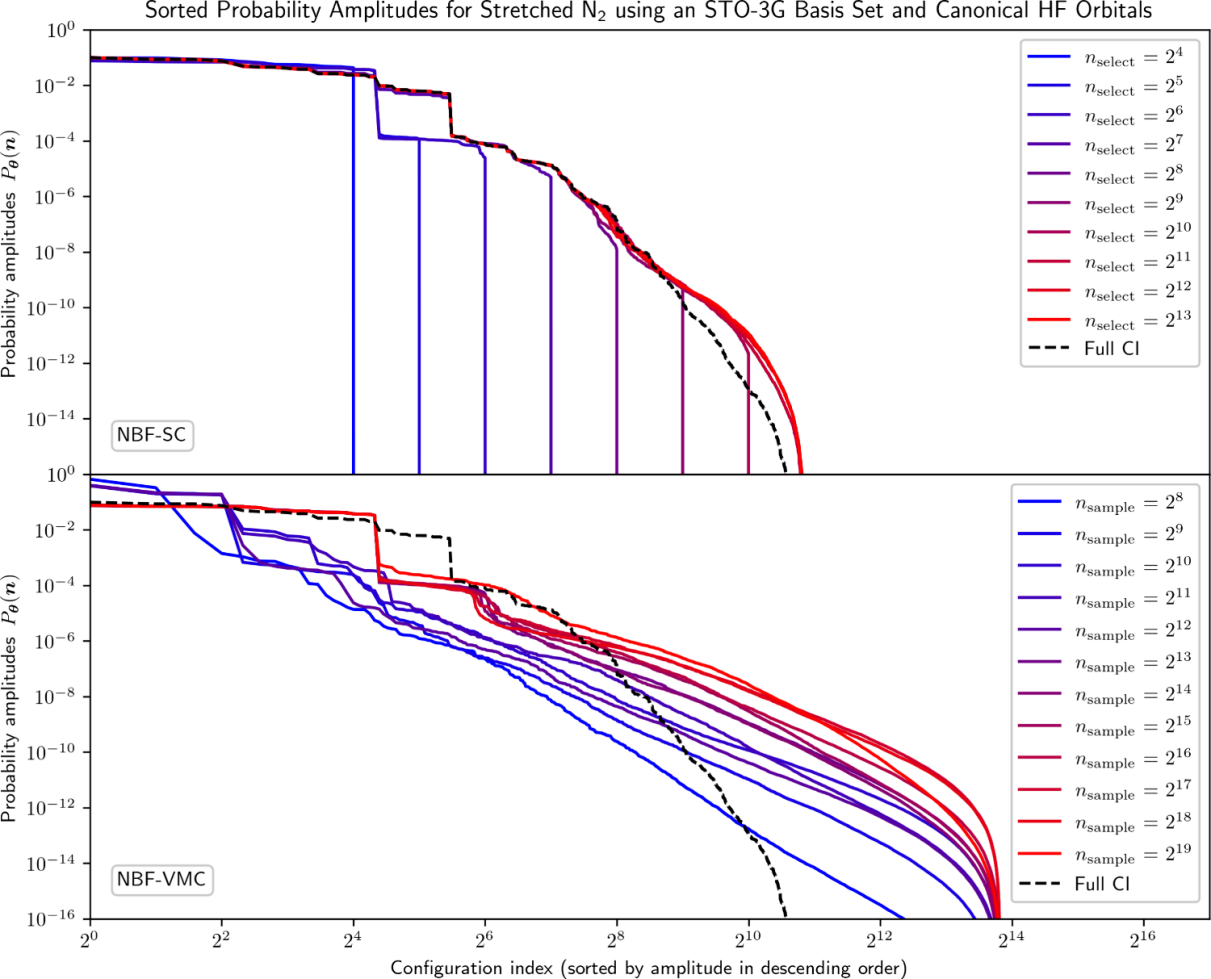
Sorted probability amplitudes from the NBF model trained with varying *n*_select_ and *n*_sample_ using selected configurations and exact Monte Carlo sampling compared to the FCI solution for stretched N_2_ using an STO-3G basis set and canonical HF orbitals (14,400 total configurations).

**Figure 2:**
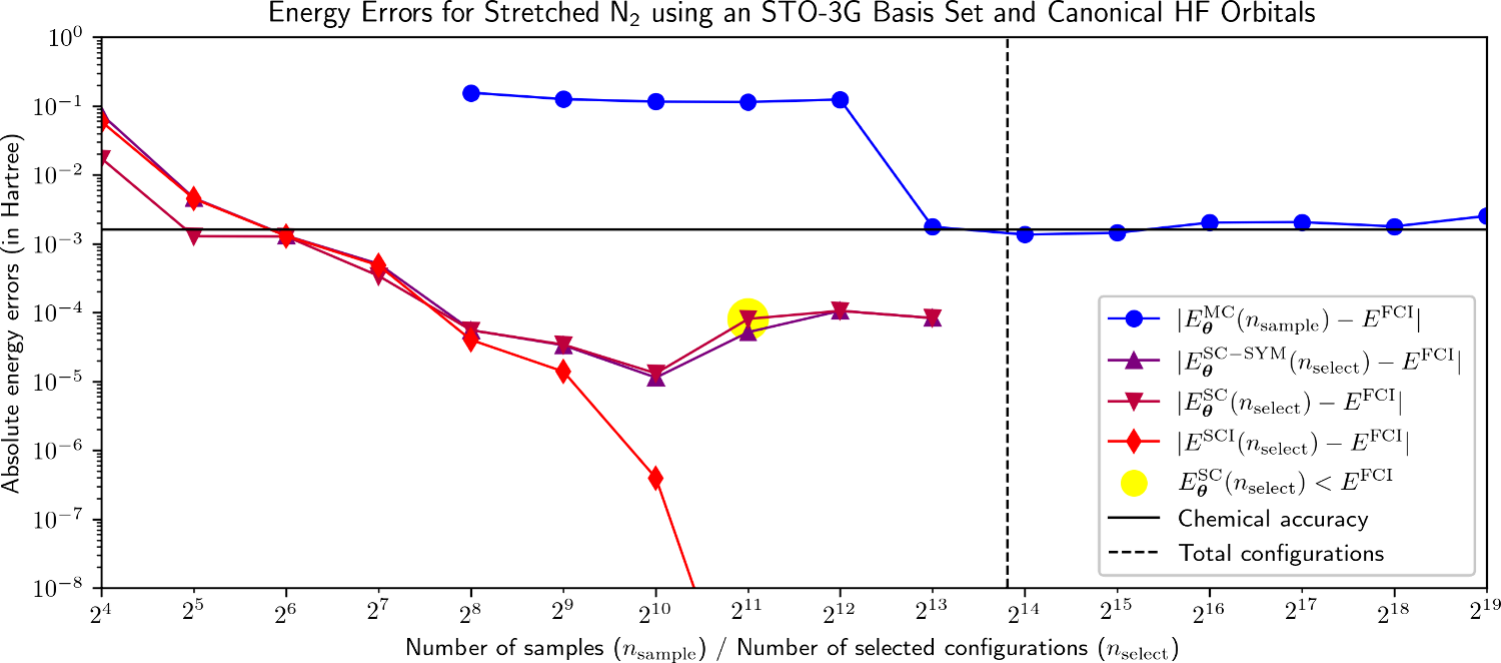
Energy errors for the NBF solutions plotted in Figure 1. For *n*_select_ = 2^11^, |*E* ^SCI^(*n*_select_) – *E* ^FCI^| drops down to approximately 5 × 10^–11^ Ha where it remains for subsequent *n*_select_.

We see that even with a small number of selected configurations, NBF-SC can effortlessly identify the 20 most dominant (largest amplitude) configurations. It struggles slightly more with the amplitude plateau induced by the 24 next most dominant configurations, but manages to identify them once *n*_select_ ≥ 2^7^. While NBF-SC can predict the highest-contributing amplitudes reasonably well, it struggles with describing the tail of the distribution (where *P*_**θ**_(***n***) ≲ 10^–8^). In terms of energies, *E*_**θ**_^SC–SYM^ drops below chemical accuracy (i.e., below an energy error of about 1.6 mHa) for *n*_select_ = 2^6^ and improves until reaching its minimum error at 11.4 μHa for *n*_select_ = 2^10^. It is particularly notable that while *E* ^SCI^ remains close to *E*_**θ**_^SC–SYM^ for *n*_select_ ≤ 2^8^, *E* ^SCI^ drops significantly below *E*_**θ**_^SC–SYM^ for *n*_select_ ≥ 2^9^ reaching an error of approximately 5 × 10^–11^ Ha ≈ 0 for *n*_select_ ≥ 2^11^. The remarkable accuracy of *E* ^SCI^ signals that NQS-SC successfully found the correct configurations, although the predicted coefficients are not highly accurate, as *E*_**θ**_^SC–SYM^ actually increases for *n*_select_ > 2^10^. It is also noteworthy that for *n*_select_ = 2^11^, *E*_**θ**_^SC^ falls below *E* ^FCI^, indicating that the nonvariationality of this energy estimate can pose an issue.

As for NBF-VMC, the 20 most dominant configuration amplitudes are only recovered once *n*_sample_ ≥ 2^13^ are considered. This sudden improvement in the predicted probability amplitudes as *n*_sample_ is increased from 2^12^ to 2^13^ corresponds to a significant drop in the energy error from 124 mHa to 1.76 mHa. Chemical accuracy is finally reached at *n*_sample_ = 2^14^ = 16,384, which is on the same order as the dimension of the Hilbert space dim(H)= 14,400. From this point on, the NBF-VMC energy error does not significantly improve any further. This behavior may be attributed to the fact that NBF-VMC never recovers the large amplitudes for the 21st to 44th most dominant configurations in the FCI solution. At the same time, NQS-VMC completely fails to describe the tail of the amplitude distribution even as *n*_sample_ is increased.

## H_2_O

The H_2_O molecule in a 6-31G basis set is taken as an example of a system with comparatively strong dynamical correlation. Comparisons of the probability amplitudes predicted by NBF-VMC and NBF-SC, as well as of the corresponding energy estimates, are provided in Figures 3 and 4, respectively.

**Figure 3:**
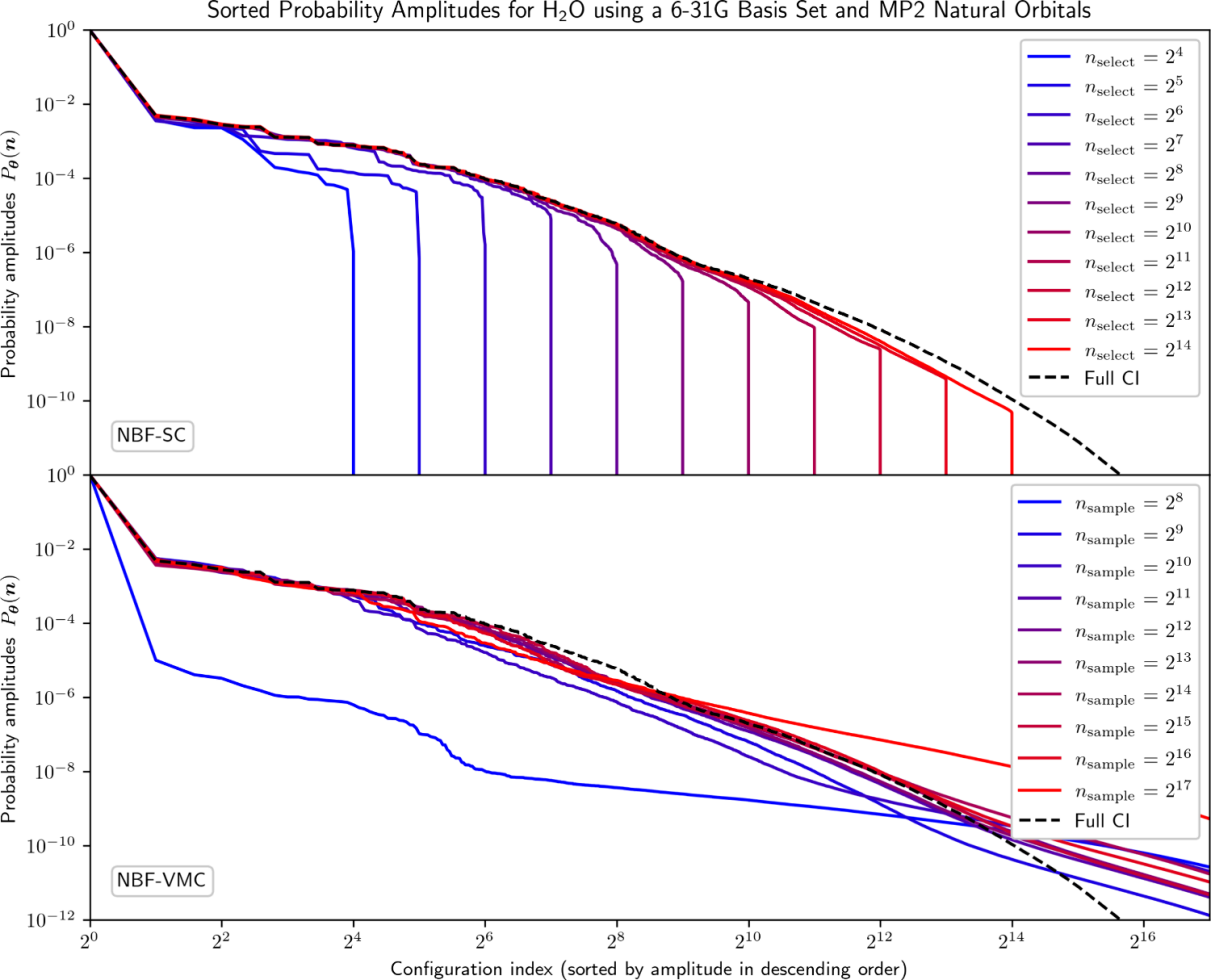
Sorted probability amplitudes from the NBF model trained with varying *n*_select_ and *n*_sample_ using selected configurations and exact Monte Carlo sampling compared to the FCI solution for H_2_O using a 6-31G basis set and MP2 natural orbitals (1,656,369 total configurations).

**Figure 4:**
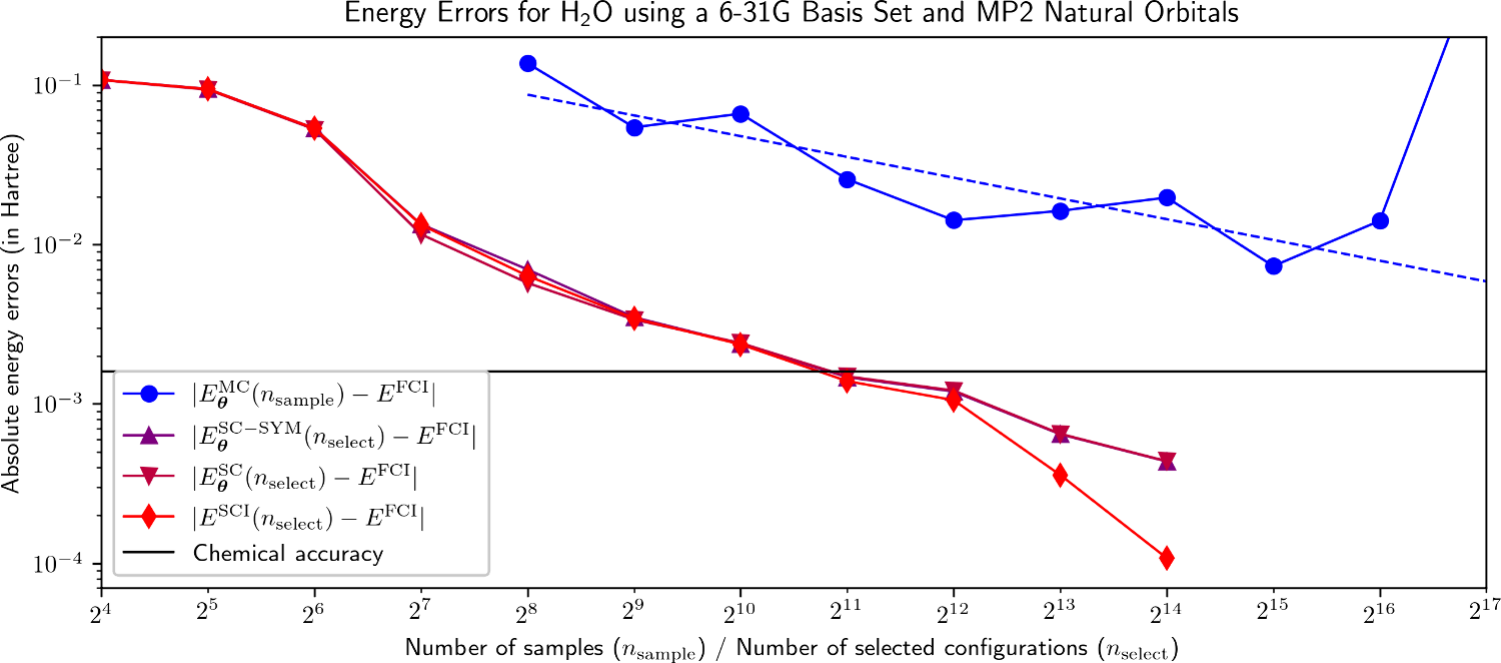
Energy errors for the NBF solutions plotted in Figure 3. |*E*_**θ**_^MC^(*n*_sample_) – *E* ^FCI^| for *n*_sample_ = 2^17^ is approximately 837 mHa. The blue dashed trendline was calculated by linear least-squares in log–log space, ignoring the outlier value of |*E*_**θ**_^MC^(*n*_sample_) – *E* ^FCI^| for *n*_sample_ = 2^17^.

In terms of coefficients, the probability amplitudes predicted by NQS-SC for a given *n*_select_ tend to start deviating earlier (at lower configuration indices) from the FCI solution for the H_2_O molecule compared to the stretched N_2_ molecule. These deviations can, however, for the most part be counteracted by simply increasing *n*_select_. In terms of energy, *E*_**θ**_^SC‑SYM^ also systematically improves as *n*_select_ is increased, and finally reaches chemical accuracy at *n*_select_ = 2^11^. Furthermore, in contrast to stretched N_2_, *E*_**θ**_^SC^ remains above *E* ^FCI^ for all calculations. Also, *E*_**θ**_^SC^ and *E*_**θ**_^SC‑SYM^ remain close for all *n*_select_, which means the asymmetric evaluation of the SC energy is not an issue here. However, the energy optimization still struggles for large *n*_select_. This is indicated by the observation that *E* ^SCI^ visibly drops below *E*_**θ**_^SC‑SYM^ for large enough *n*_select_, meaning the wave function coefficients predicted by NQS-SC are not optimal for that selected subspace. Overall, Figures 3 and 4 show that the NQS-SC procedure performs reasonably well for systems dominated by dynamical correlation, with systematic improvements in the energy errors, but also at the cost of an increasingly large number of selected configurations, as one would expect.

NBF-VMC struggles even more with H_2_O compared to stretched N_2_. Although the energy error now steadily improves with more samples, it never reaches chemical accuracy. For *n*_sample_ = 2^17^, the VMC calculations do not even converge, which is not uncommon and presumably due to suboptimal sample batches pushing the model off its convergence trajectory. Changing the sampler seed restores the expected convergence behavior. With a linear regression in log–log space while ignoring the outlier for *n*_sample_ = 2^17^, we extrapolate *E*_**θ**_^MC^ to fall below chemical accuracy for *n*_sample_ ≈ 2,643,513 ≈ 2^21.3^; that is, for NBF-VMC to reach chemical accuracy, it would again take *n*_sample_ on the order of the dimension of the entire Hilbert space dim(H)= 1,656,369. This is contrary to the conceptual advantage that NQS-VMC should have over NQS-SC, namely that, as an approach operating over the entirety of H , NQS-VMC should be able to better capture dynamical correlation than NQS-SC. In practice, the minimum error obtained with NBF-SC in the presence of dynamical correlation remains over an order of magnitude lower than that achieved by NBF-VMC.

We have carried out the NBF-SC calculations nine additional times for the stretched N_2_ and the H_2_O molecule in a 6-31G basis set for *n*_select_ = 2^6^ and *n*_select_ = 2^11^, respectively, i.e., for the smallest *n*_select_ values for which chemical accuracy was reached. Every rerun used a different random seed through which the $S_{\mathrm{expand}}\subset S_{\mathrm{extend}}$ were selected. The corresponding results are, for stretched N_2_, |*E*_**θ**_^SC–SYM^(2^6^) – *E* ^FCI^| = 1.590 ± 0.289 mHa and, for H_2_O, |*E*_**θ**_^SC–SYM^(2^11^) – *E* ^FCI^| = 1.453 ± 0.083 mHa, where the numbers after the ± signs represent 95*%* confidence intervals (1.96 times the standard error of the mean).

In addition to the two example systems discussed above, we extend our analysis of NQS-VMC and NQS-SC to N_2_ at equilibrium bond length (1.0976 Å), LiCl, C_2_H_4_, and Li_2_O, using experimental geometries from ref 52 and a combination of STO-3G, 6-31G, and 6-311G basis sets (see Table 1). Probability amplitude profiles and energies similar to those presented in Figures 1–4 are provided for all these systems in the Supporting Information. For these additional systems, NBF-SC again shows a consistent systematic improvement with increasing *n*_select_. By contrast, the optimized energies of NBF-VMC experienced either sudden and nonsystematic jumps with increasing *n*_sample_ or a too slow convergence (often accompanied by sporadic nonconvergence) to chemical accuracy. In addition, we observe that *E*_**θ**_^SC^ only drops below *E* ^FCI^ in the case of one additional system besides stretched N_2_, namely for LiCl for *n*_select_ = 2^7^. This indicates that the nonvariationality of *E*_**θ**_^SC^ only manifests itself rarely.

**Table 1:** Overview of the Minimal (Power-of-Two) *n*_select_ Values Needed for NBF-SC to Reach Chemical Accuracy (|*E*_***θ***_^SC–SYM^(*n*_select_) – *E* ^FCI^| < 1.6 mHa) for Various Combinations of Molecules and Basis Sets^a^

Molecule	Basis set	dim (H)	*n*_select_	$n_{\mathrm{select}}/\dim\left(H\right)$
N_2_	STO-3G	14,400	2^7^	0.889*%*
Stretched N_2_	STO-3G	14,400	2^6^	0.444*%*
LiCl	STO-3G	1,002,001	2^7^	0.013*%*
H_2_O	6-31G	1,656,369	2^11^	0.124*%*
C_2_H_4_	STO-3G	9,018,009	2^13^	0.091*%*
Li_2_O	STO-3G	41,409,225	2^10^	0.002*%*
H_2_O^a^	6-311G^a^	135,210,384	2^14.14^ ^a^	0.013*%* ^a^

a The result for the H_2_O molecule in a 6-311G basis set was found by linear extrapolation in log–log space, as 2^13^ was the largest *n*_select_ possible within NetKet before running out of hardware memory resources. Much more efficient energy evaluations (among other improvements) are possible in principle, but would require major changes in NetKet or writing a new NQS program from scratch.

b dim(H) provides the total dimensionality of the underlying Hilbert spaces, while $n_{\mathrm{select}}/\dim\left(H\right)$ denotes the percentage of total configurations that had to be selected to reach chemical accuracy.

In Table 1 we present an overview of the minimal *n*_select_ required for NBF-SC to reach chemical accuracy for all molecules, except for H_2_O using a 6-311G basis set, where the minimal required *n*_select_ was obtained via extrapolation. Overall, NQS-SC consistently required fewer than 1*%* of all configurations to be selected to reach chemical accuracy. Remarkably, the minimal *n*_select_ for stretched N_2_ is half of that for N_2_ at its equilibrium bond length, despite the fact that the former is more strongly correlated. This confirms that NQS-SC is better suited for systems dominated by static rather than dynamical correlation. Furthermore, enlarging the basis set for H_2_O from 6-31G to 6-311G reduces the ratio $n_{\mathrm{select}}/\dim\left(H\right)$ by a factor of 10. While this is generally reassuring for the scalability of NQS-SC with basis set size, the rate of decay of this ratio is still too slow so that, with a 6-311G basis set, chemical accuracy could not be reached before hardware memory constraints were hit. However, we note that this limitation is rather a consequence of the current energy evaluation in NetKet, although the approach also suffers from the general drawbacks of stand-alone SCI methods, demanding more configurations in such cases. This further underscores NQS-SC’s susceptibility to systems with slowly decaying amplitude distributions.

## Stretched H_8_

As a final challenge, we consider the stretched hydrogen chain H_8_ with a bond length of 1.8 Å in a minimal STO-3G basis set. This system is characterized by an extremely slowly decaying amplitude distribution, blurring the boundary between static and dynamical correlation. The probability amplitudes predicted and the energy errors incurred by NBF-SC and NBF-VMC for this system are plotted in Figures 5 and 6, respectively.

**Figure 5:**
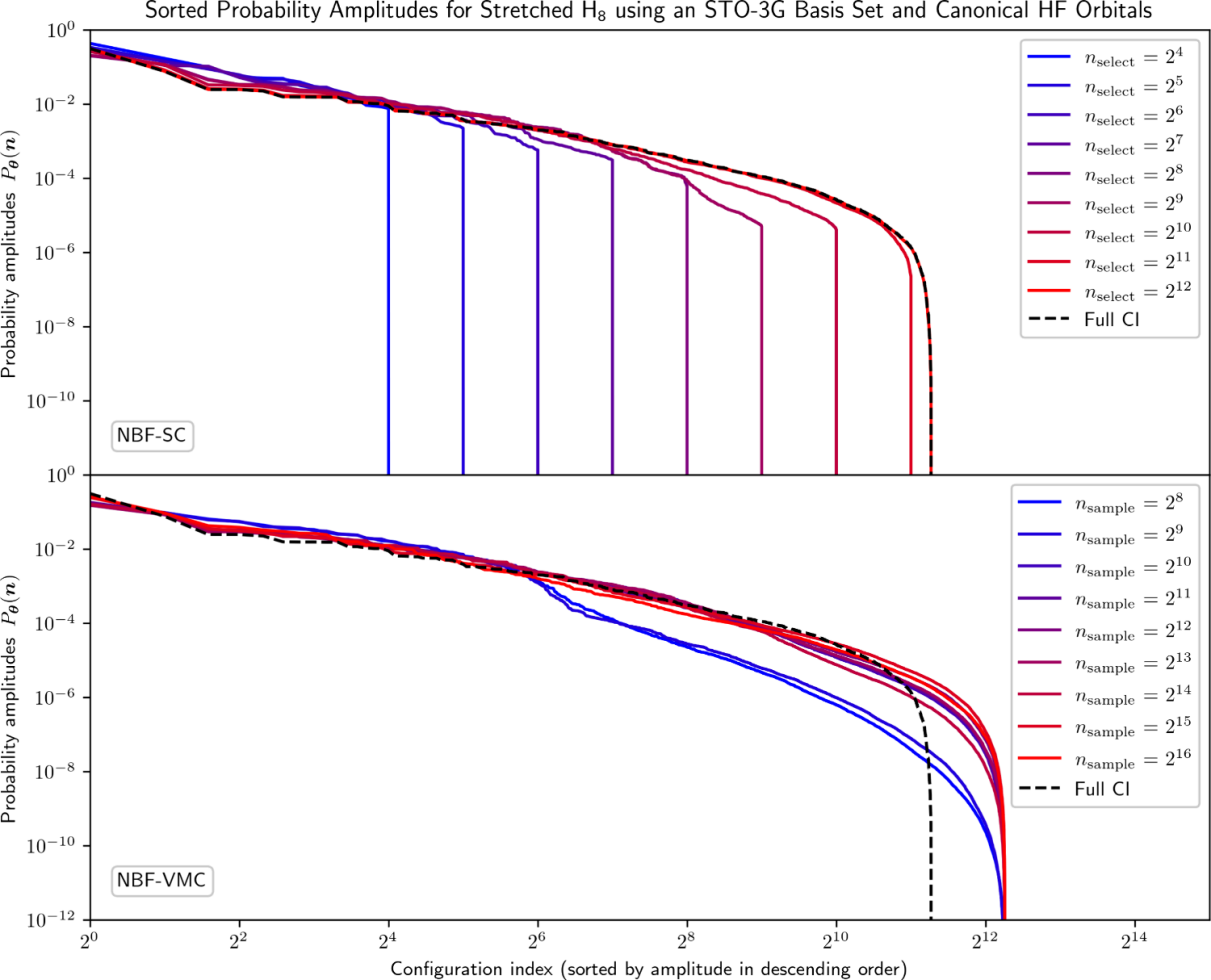
Sorted probability amplitudes from the NBF model trained with varying *n*_select_ and *n*_sample_ using selected configurations and exact Monte Carlo sampling compared to the FCI solution for stretched H_8_ using an STO-3G basis set and canonical HF orbitals (4,900 total configurations).

**Figure 6:**
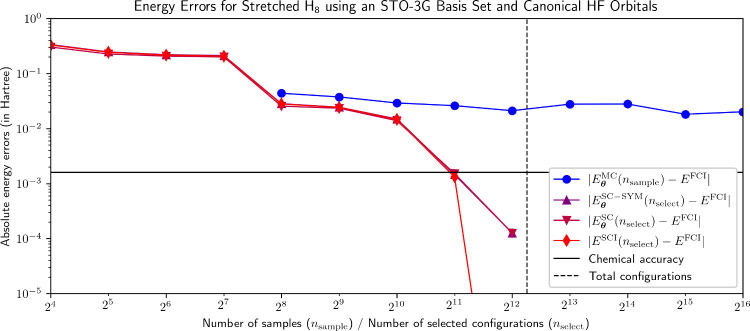
Energy errors for the NBF solutions plotted in Figure 5. For *n*_select_ = 2^12^, |*E* ^SCI^(*n*_select_) – *E* ^FCI^| drops down to approximately 1.25 × 10^–10^ Ha.

NBF-SC convergence with increasing *n*_select_ is less smooth than it was for the previously examined systems. There are two major regimes in which significant reductions in the energy error are achieved: from *n*_select_ = 2^7^ to *n*_select_ = 2^8^ and from *n*_select_ = 2^10^ onward. In the latter case, this corresponds to NBF-SC successfully identifying sets of configurations that enable it to match the FCI amplitudes almost perfectly. Even so, for *n*_select_ = 2^11^, both *E*_**θ**_^SC–SYM^ and *E* ^SCI^ barely drop below the chemical accuracy threshold, illustrating the fact that a substantial fraction of total configurations is required for an SCI-type wave function to accurately describe this system. Nevertheless, both selecting the correct configurations and assigning them accurate wave function coefficients are highly nontrivial tasks accomplished by the NQS-SC framework.

NBF-VMC, by contrast, starts at a relatively low initial energy error, yet fails to materially reduce this error for increasing *n*_sample_. Its predicted amplitude spectrum noticeably improves when transitioning from *n*_sample_ = 2^9^ to *n*_sample_ = 2^10^, with a corresponding energy error reduction from 37.5 mHa to 29.2 mHa. Yet, NBF-VMC fails to reproduce the tail of the FCI amplitude distribution, regardless of the number of samples used, while also never even approaching chemical accuracy, to the extent that even extrapolating an *n*_sample_ (to reach chemical accuracy) appears to be impossible.

In this work, we opted for delocalized molecular orbitals to represent the wave functions. Unlike for real-space NQS, the choice of orbital basis can drastically affect the ground state’s correlation pattern in a second-quantized setting. A systematic investigation of the impact of the orbital basis on the performance of either NQS-VMC or NQS-SC would be an interesting direction for future work. That being said, the present results for stretched N_2_ and stretched H_8_ already indicate that an increased multireference character (which would be found for localized orbitals) does not favor NQS-VMC over NQS-SC.

To summarize, we compared the performance of two NQS frameworks, NQS-VMC and NQS-SC, in the context of *ab initio* quantum chemistry. We found that, despite the dominance of NQS-VMC in the literature, NQS-SC is a more reliable method in terms of both accuracy and systematic improvability. Our detailed analysis of wave function coefficient profiles obtained with both frameworks demonstrates that NQS-SC can efficiently capture static correlation in electronic ground states and, to some extent, dynamical correlation.

By contrast, NQS-VMC generally struggles to incorporate either type of correlation. Both calculated and extrapolated data indicate that NQS-VMC tends to require an enormous number of samples, rivaling or even exceeding the Hilbert space’s total dimension to reach chemical accuracy. However, a large selected configuration space is also needed for NQS-SC to describe molecules dominated by dynamical correlation, although the scaling appears to be far less severe. In such regimes, NQS-SC is unlikely to be a competitive method, as dynamical correlation without its static counterpart can be reasonably accounted for by single-reference methods, such as those based on coupled cluster theory.^53,54^

Even for the extremely strongly correlated stretched H_8_ chain, we found that, given a sufficiently large selected space, NQS-SC can achieve chemical accuracy, showcasing its expressiveness. Although, due to the very flat amplitude distribution of this system, NBF-SC’s selected space must contain a large fraction of total configurations in B . This illustrates that, similar to traditional SCI approaches, NQS-SC’s efficient applicability is reliant on the multiconfigurational character within the system being confined to a limited subset of total configurations. Fortunately, many chemical systems of interest with multiconfigurational character, particularly transition states, fall within this category (see the class-1 cases in ref 55), thereby offering a wide domain of applicability for NQS-SC. By contrast, the class-2 cases in ref 55 will pose challenges for SCI and hence also NQS-SC. Nevertheless, NQS-SC offers a conceptually distinct and potentially self-consistent method for subspace selection, using wave function coefficients predicted without subspace diagonalization. Therefore, an important open question for future research is whether NQS can be leveraged to approach energetically optimal subspaces, which remain largely out of reach for most problems.

Since NQS are already known to represent highly entangled lattice wave functions efficiently, their current limitations in quantum chemistry arise from practical challenges, such as network optimization and energy evaluation, rather than expressiveness. Addressing these bottlenecks will be necessary to enable NQS to compete with state-of-the-art approaches such as SCI and DMRG. The present work shall be understood as an important step in this direction.

Our results highlight a key aspect of NQS development: how information, such as energy and gradients, is extracted from the neural network. The default VMC framework fundamentally limits the efficiency and applicability of NQS to large-scale electronic structure calculations, especially in the regime of interest where both static and dynamical correlation are crucial for accurate solutions. Such a limitation is independent of the choice of the Monte Carlo sampling method, as we have conducted our comparison using an exact sampling scheme. As such, we anticipate that NQS-SC will emerge as the new default approach for future NQS implementations for electronic structure calculations. Our findings also underscore the importance of developing improved techniques for extracting physical information from NQS, an aspect that has received limited attention to date.

With more advanced neural network architectures for predicting electronic wave functions now available, it is worthwhile to take a step back and re-evaluate the orthogonal aspect of information extraction to maximize the benefits of these advancements. Another potential route to scale up NQS is to confine them to an active space. This approach is already common for strongly but primarily statically correlated ansätze such as matrix product states,^56,57^ and also for quantum computing, where computational resources are limited.^58,59^ For these, the missing dynamical correlation is usually recovered through the subsequent application of a perturbation (see, e.g., refs 60–64 ) or coupled cluster theory.^65–69^ In this context, NQS-SC naturally emerges as a suitable foundation for future hybrid static–dynamical methods.

## Supplementary Material



## Data Availability

The data and source code used to produce the results of this work are available on Zenodo.^70^

## Notes

Methodological details for the calculations featured in this Letter; Supplementary plots for the molecular systems featured in Table 1 (PDF)
