# Supplementary material for: Neural Quantum States Based on Selected Configurations
Source: J Phys Chem Lett. 2026 Apr 23;17(18):5180–90. doi: 10.1021/acs.jpclett.6c00520 (PMC13158996; doi:10.1021/acs.jpclett.6c00520)
Supplement: Supplementary file 1 [file jz6c00520_si_001.pdf]

Supporting Information for "Neural Quantum States Based on Selected Configurations"

Marco Julian Solanki, Lexin Ding, and Markus Reiher*

ETH Zürich, Department of Chemistry and Applied Biosciences, Vladimir-Prelog-Weg 2, CH-8093 Zürich, Switzerland

E-mail: mreiher@ethz.ch

Methological Details

Machine Learning Considerations

Neural backflow (NBF) models with two feedforward layers were considered ($K = 2$). The width of the single hidden layer was fixed to be $4L$, i.e. $\boldsymbol{W}^{(1)} \in \mathbb{R}^{4L\times 2L}$ and $\boldsymbol{b}^{(1)} \in \mathbb{R}^{4L}$. The activation function $\boldsymbol{\sigma}$ was fixed to be a hyperbolic tangent function $\tanh(\boldsymbol{x})$, which is understood to be taken element-wise over $\boldsymbol{x}$. A single backflow determinant ($D = 1$) was used for all systems apart from H_2O with a 6-311G basis set, for which $D = 2$ was used, as it seemed to help getting (close to) chemical accuracy for the considered n_{select}. The model's learnable parameters, implemented as 32-bit floating-point numbers, were initialized using Lecun normal initialization.

All experiments were performed using the Adam optimizer[1] with constant learning rates of $5 \cdot 10^{-3}$ (selected configurations) / $2 \cdot 10^{-3}$ (exact Monte Carlo sampling). A reduced learning rate for exact Monte Carlo sampling was chosen to reduce the number of cases in which the model would "jump out" of local energy minima it had already discovered. All experiments were run for 10,000 training iterations.

For selected configurations, n_{expand} was chosen to be $n_{\text{select}} / 8$, apart from for the stretched hydrogen chain H_8, for which an n_{expand} value of $n_{\text{select}} / 2$ was found to yield better results.

Soft- and Hardware Setup

The calculations presented here were performed using Python 3.13.0. Extensive use was made of the NetKet library for neural quantum states (version 3.21.0).[2,3] NetKet itself relies on the JAX machine learning framework (version 0.8.2)[4] and the Flax library (version 0.12.2),[5] which builds on top of it. Hartree–Fock calculations, MP2 calculations, and reference FCI calculations were performed using the PySCF quantum chemistry package (ver-

sion 2.11.0).[6–8] Exact diagonalization on subspaces spanned by selected configurations and explicit energy expectation value evaluations were performed using the PyCI library for arbitrary determinant CI (version 0.6.4).[9]

Exact Monte Carlo sampling was performed using NetKet's integrated `ExactSampler` while selected configuration-based training was achieved through a custom re-implementation of NetKet's `MCState` class.

All calculations were run on ETH Zürich's Euler cluster on a mixture of individual Nvidia GeForce RTX 4090 & Nvidia A100 PCIe 40 GB GPUs, subject to availability constraints.

External Data Sources

Apart from the stretched nitrogen molecule, all molecular structures are experimental and retrieved from the Computational Chemistry Comparison and Benchmark Database (CCCDBD).[10]

Supplementary Plots

Figures 1–7 contain plots of the probability amplitudes for various molecular systems as predicted by the neural backflow (NBF) model. Corresponding plots of the energy errors are provided in Figures 8–14.

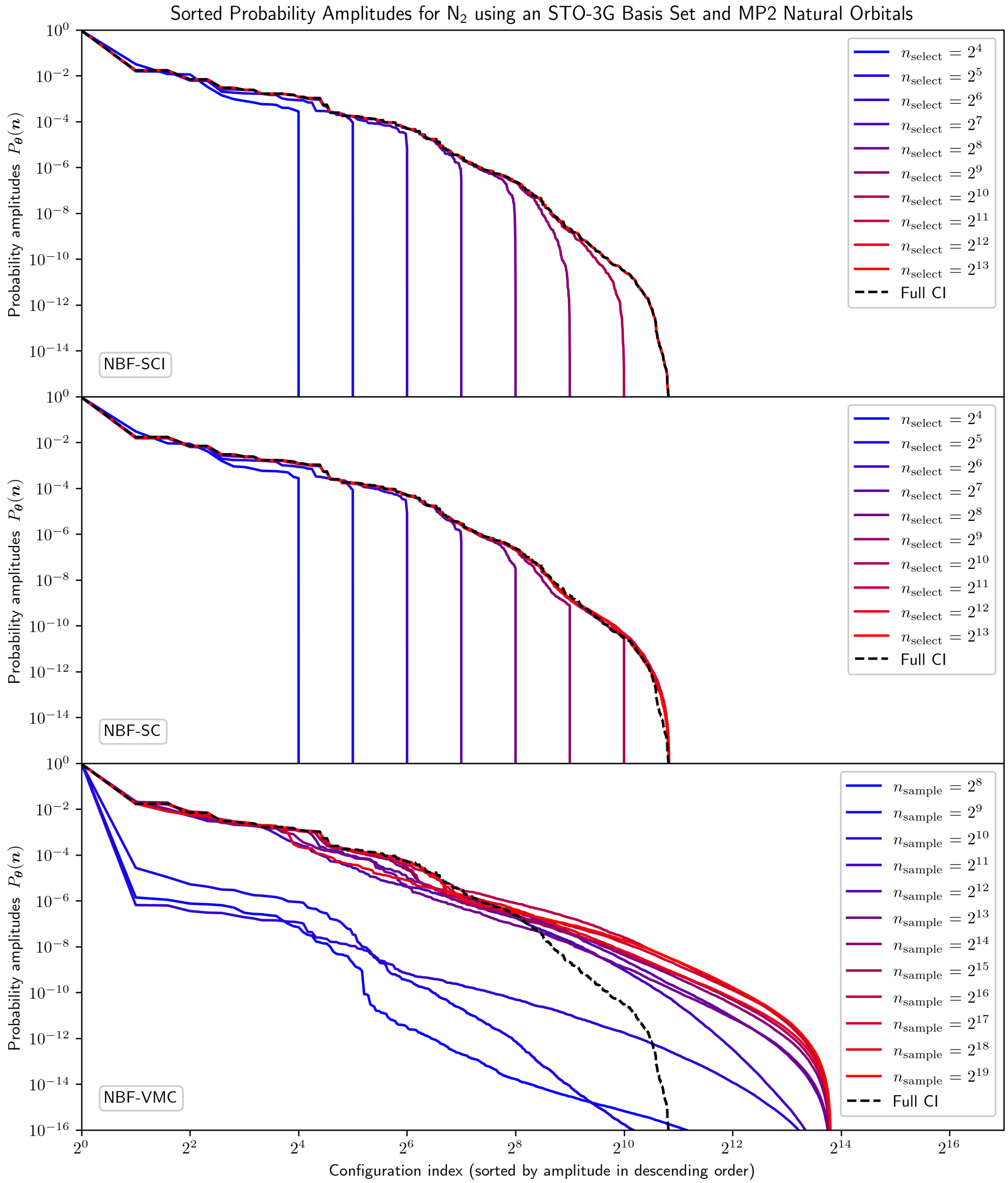

Figure 1: Sorted probability amplitudes predicted by the NBF model trained with varying n_{select} & n_{samples} using selected configurations (NBF-SC) & exact Monte Carlo sampling (NBF-VMC) compared to the FCI solution. The exact diagonalization results for the configurations selected by NBF-SC are also plotted (NBF-SCI). The system under consideration is the N_2 molecule using an STO-3G basis set and MP2 natural orbitals (14,400 total configurations).

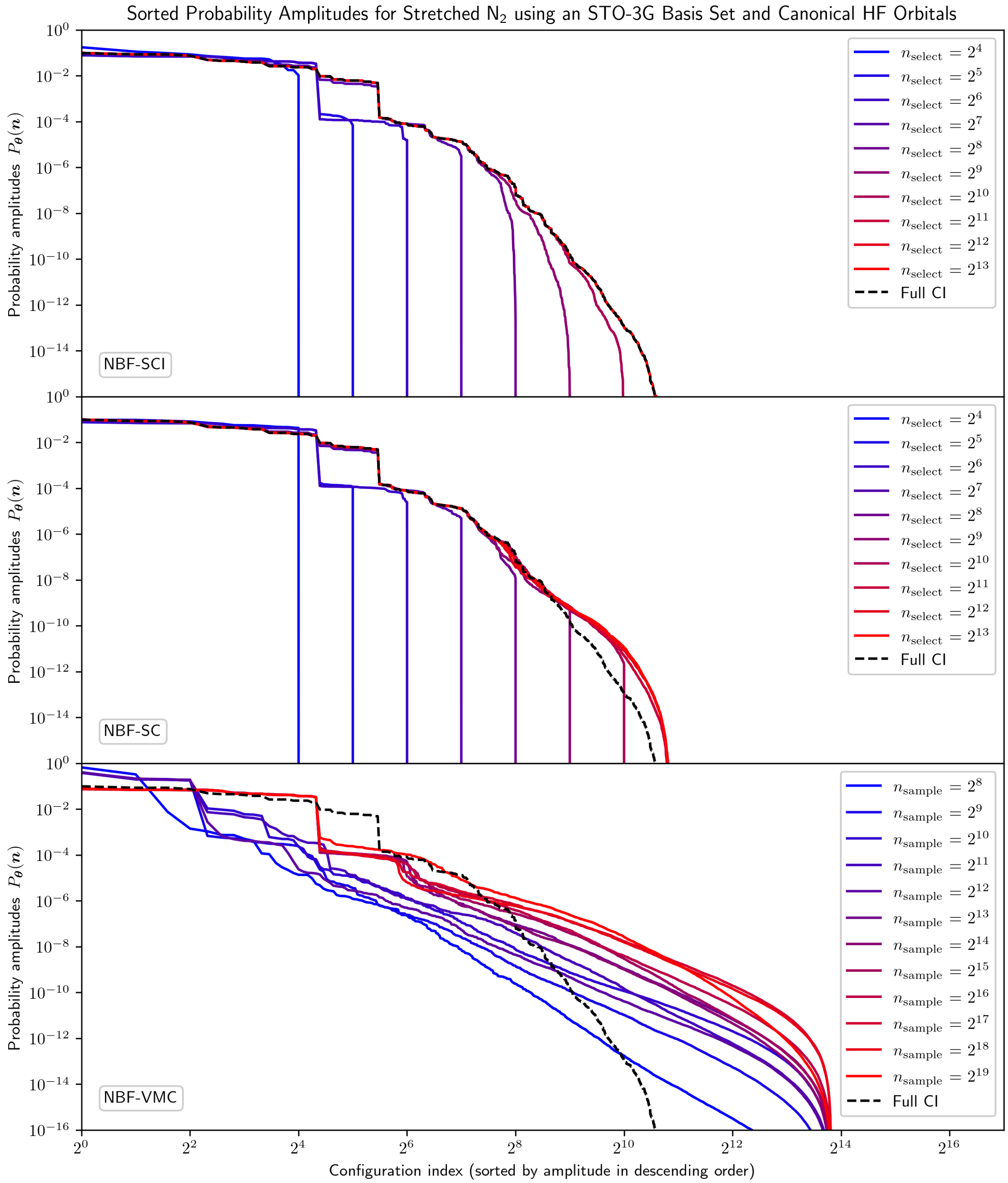

Figure 2: Sorted probability amplitudes predicted by the NBF model trained with varying n_{select} & n_{samples} using selected configurations (NBF-SC) & exact Monte Carlo sampling (NBF-VMC) compared to the FCI solution. The exact diagonalization results for the configurations selected by NBF-SC are also plotted (NBF-SCI). The system under consideration is the stretched N_2 molecule using an STO-3G basis set and canonical HF orbitals (14,400 total configurations).

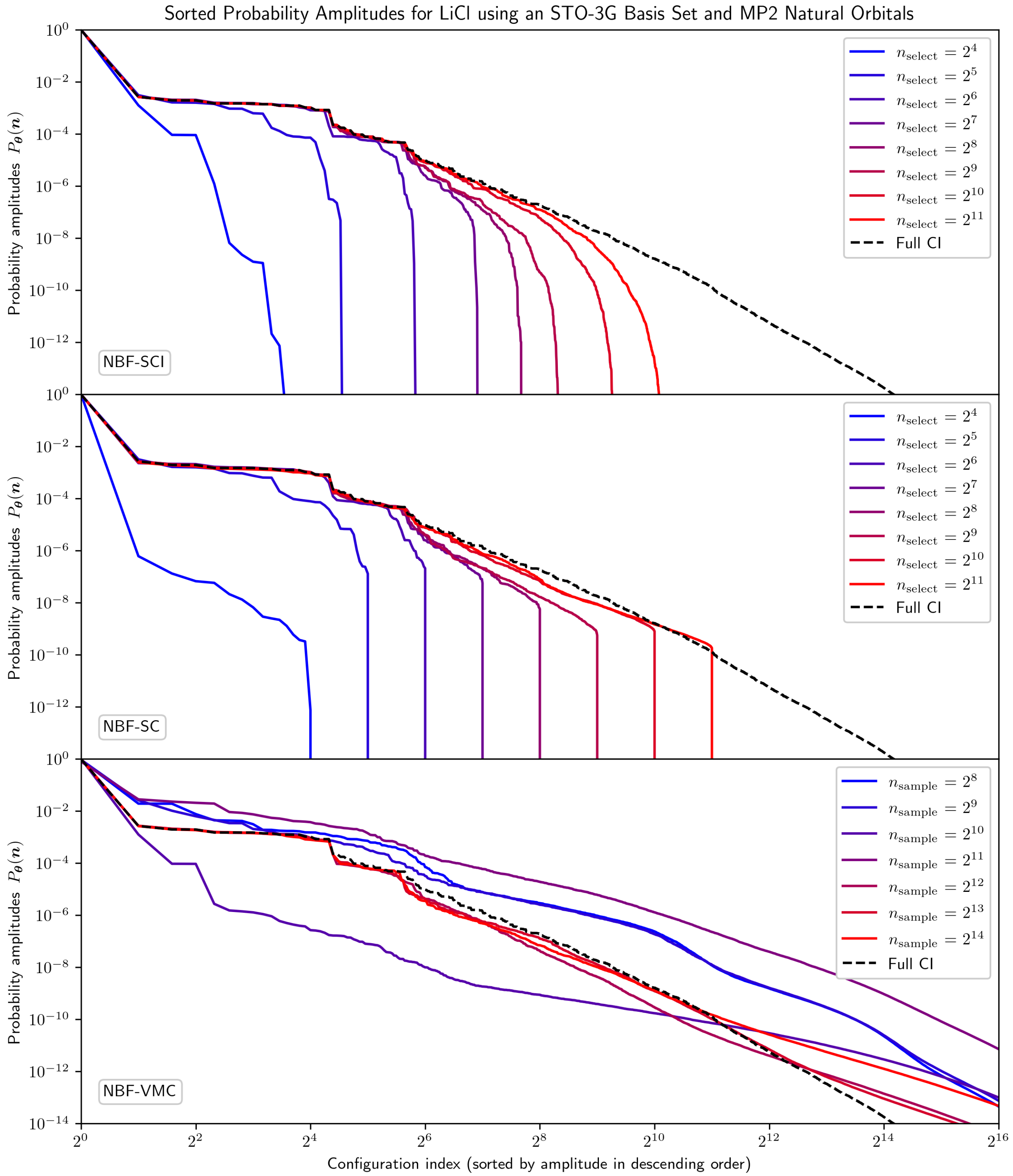

Figure 3: Sorted probability amplitudes predicted by the NBF model trained with varying n_{select} & n_{samples} using selected configurations (NBF-SC) & exact Monte Carlo sampling (NBF-VMC) compared to the FCI solution. The exact diagonalization results for the configurations selected by NBF-SC are also plotted (NBF-SCI). The system under consideration is the LiCl molecule using an STO-3G basis set and MP2 natural orbitals (1,002,001 total configurations).

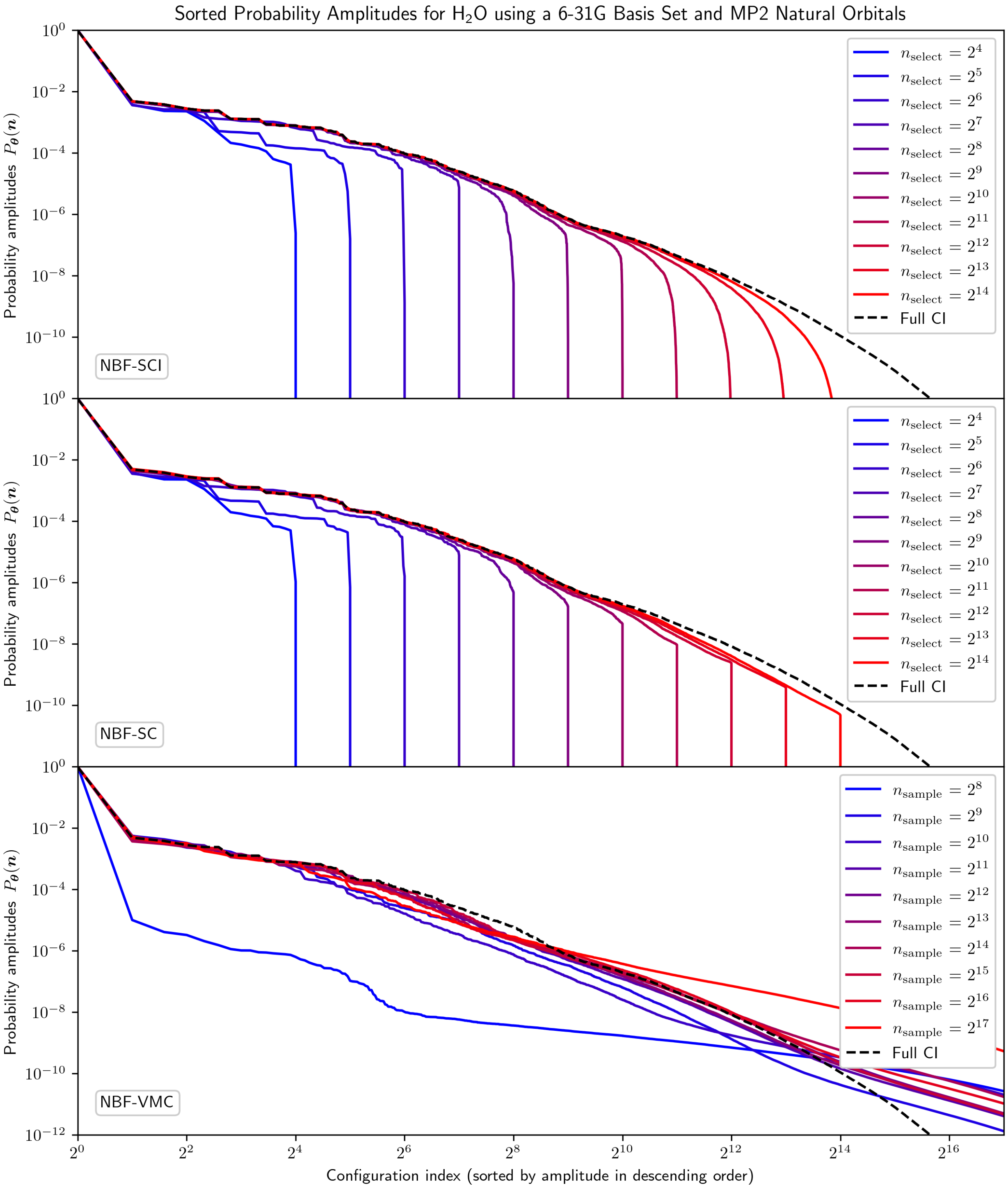

Figure 4: Sorted probability amplitudes predicted by the NBF model trained with varying n_{select} & n_{samples} using selected configurations (NBF-SC) & exact Monte Carlo sampling (NBF-VMC) compared to the FCI solution. The exact diagonalization results for the configurations selected by NBF-SC are also plotted (NBF-SCI). The system under consideration is the H_2O molecule using a 6-31G basis set and MP2 natural orbitals (1,656,369 total configurations).

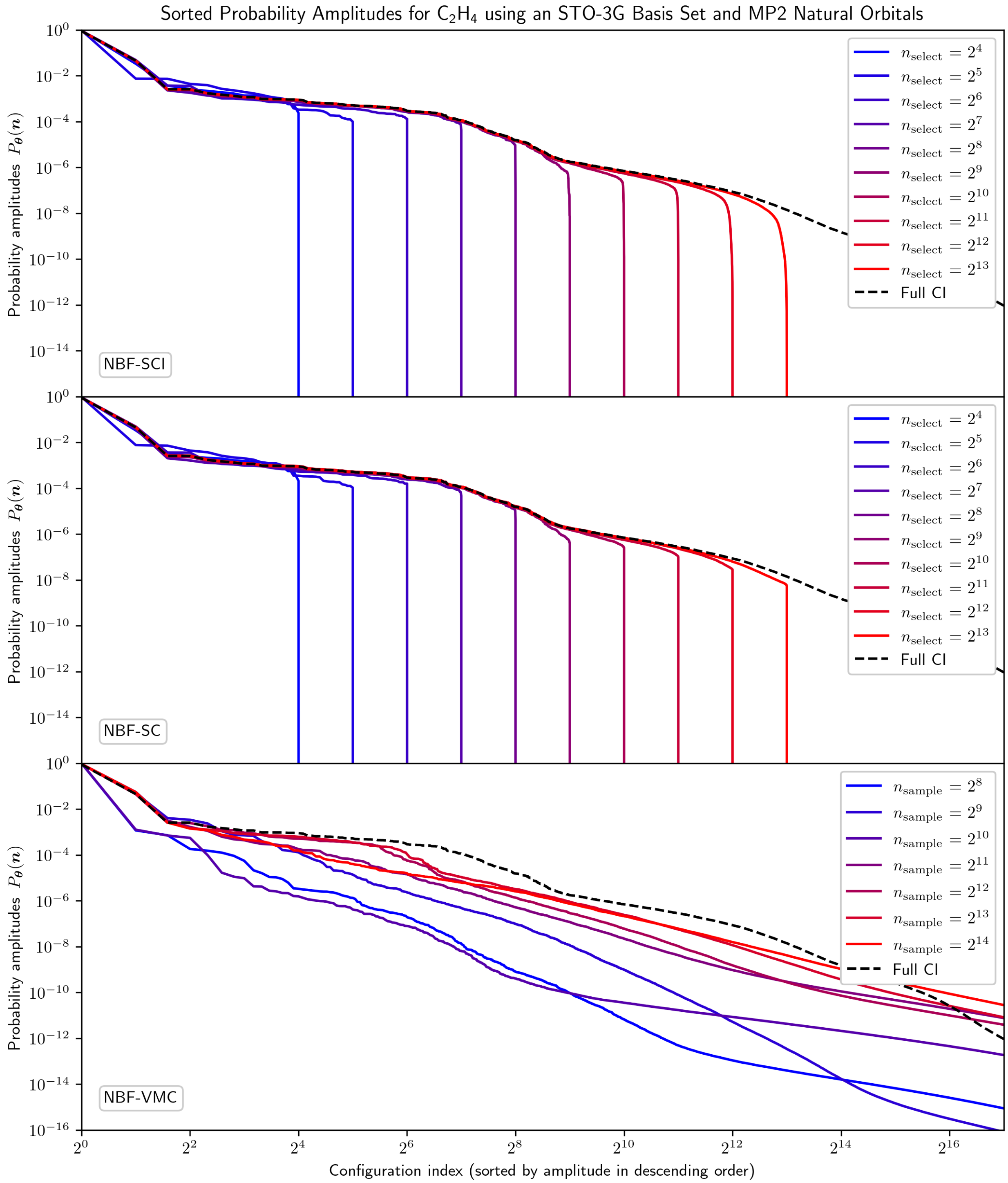

Figure 5: Sorted probability amplitudes predicted by the NBF model trained with varying n_{select} & n_{samples} using selected configurations (NBF-SC) & exact Monte Carlo sampling (NBF-VMC) compared to the FCI solution. The exact diagonalization results for the configurations selected by NBF-SC are also plotted (NBF-SCI). The system under consideration is the C_2H_4 molecule using an STO-3G basis set and MP2 natural orbitals (9,018,009 total configurations).

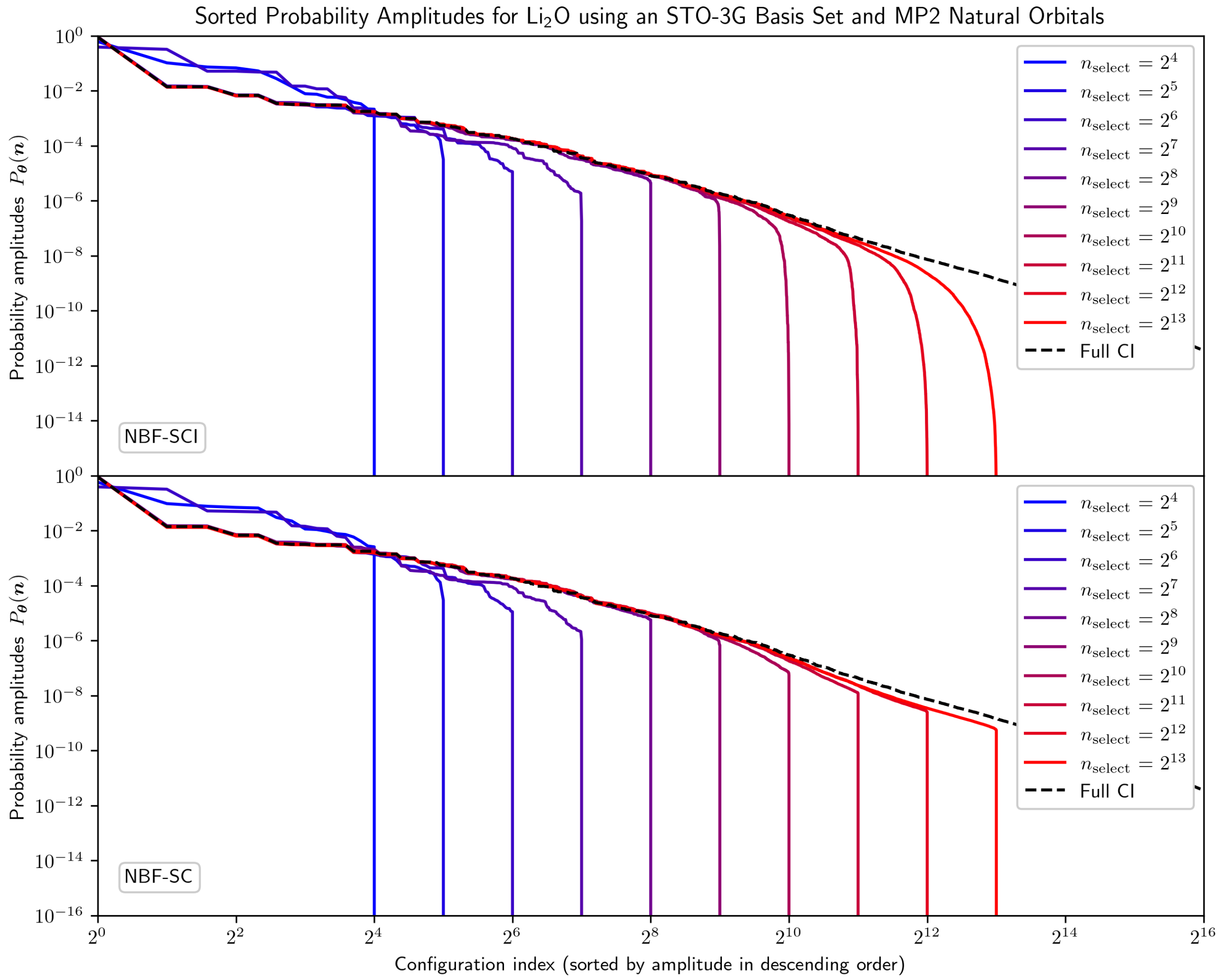

Figure 6: Sorted probability amplitudes predicted by the NBF model trained with varying n_{select} using selected configurations (NBF-SC) compared to the FCI solution. The exact diagonalization results for the configurations selected by NBF-SC are also plotted (NBF-SCI). The system under consideration is the Li_2O molecule using an STO-3G basis set and MP2 natural orbitals (41,409,225 total configurations).

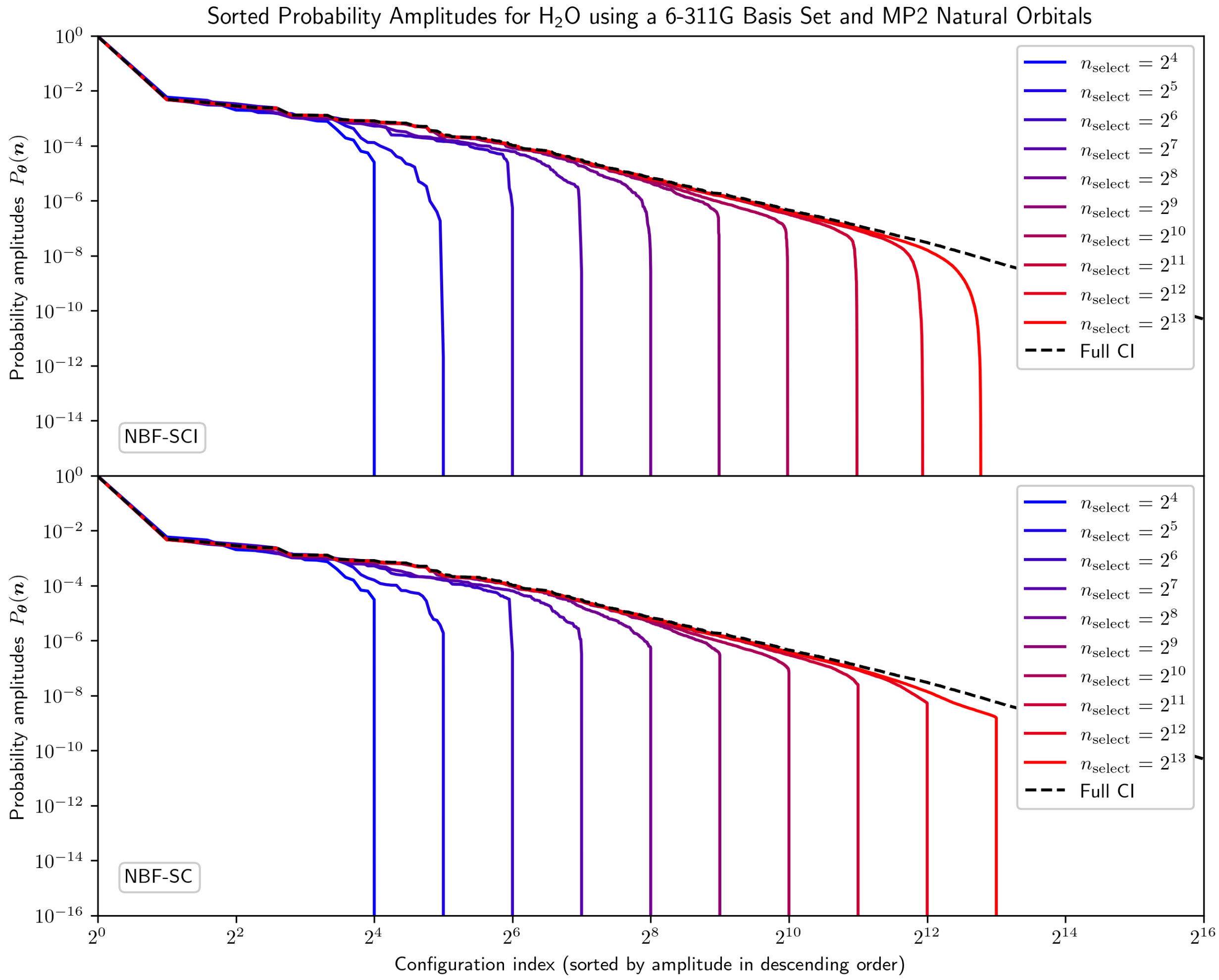

Figure 7: Sorted probability amplitudes predicted by the NBF model trained with varying n_{select} using selected configurations (NBF-SC) compared to the FCI solution. The exact diagonalization results for the configurations selected by NBF-SC are also plotted (NBF-SCI). The system under consideration is the H_2O molecule using a 6-311G basis set and MP2 natural orbitals (135,210,384 total configurations).

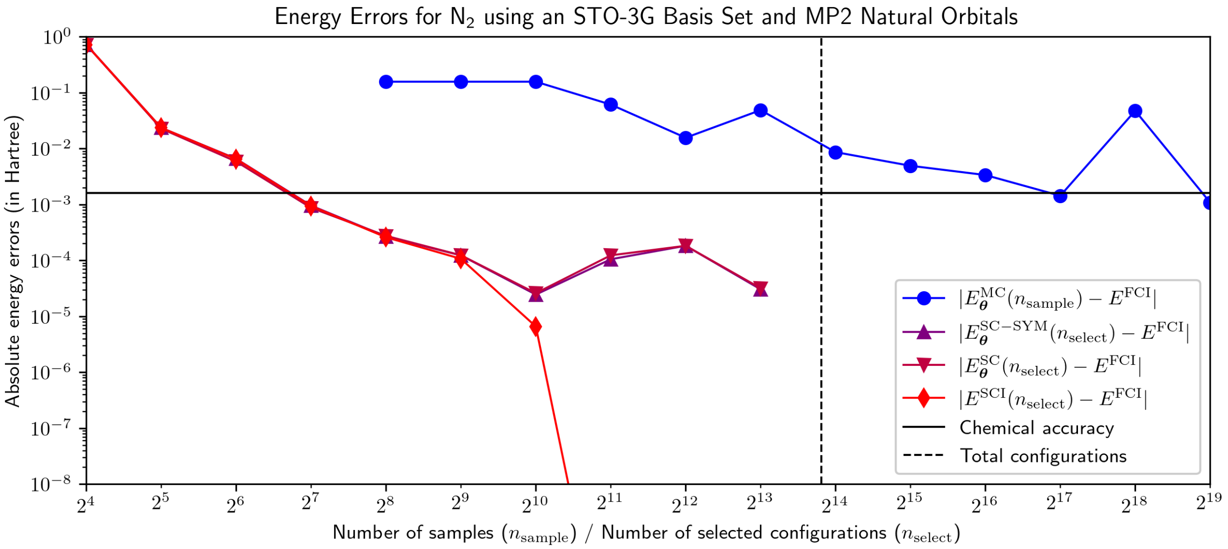

Figure 8: Energy errors for the NBF solutions plotted in Figure 1. For $n_{\mathrm{select}} = 2^{11}$, $|E^{\mathrm{SCI}}(n_{\mathrm{select}}) - E^{\mathrm{FCI}}|$ drops down to approx. $2 \cdot 10^{-12}$ Ha where it remains for subsequent n_{select}.

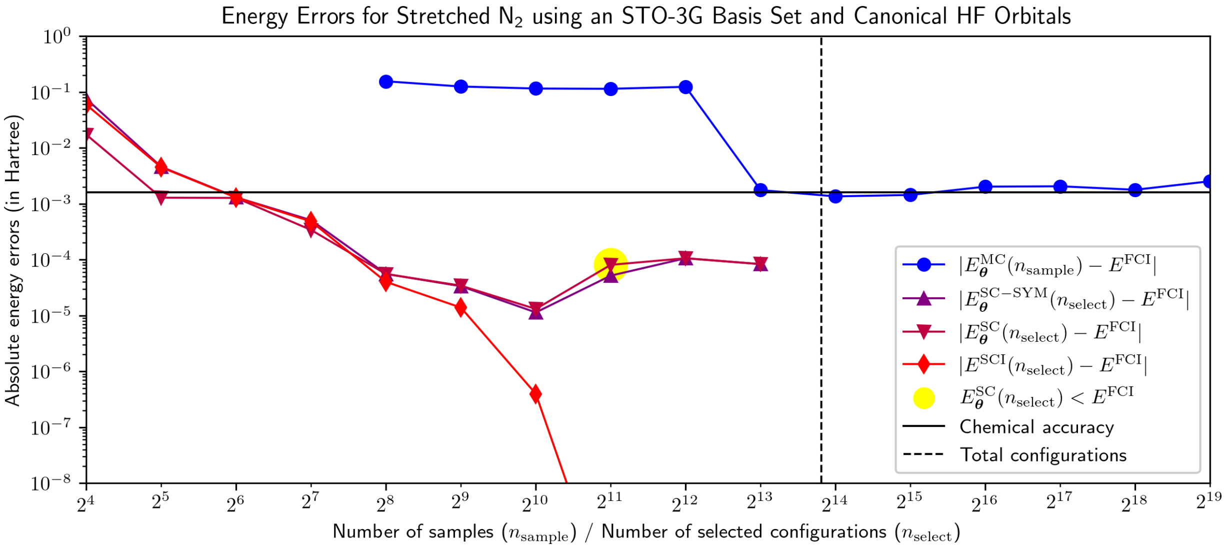

Figure 9: Energy errors for the NBF solutions plotted in Figure 2. For $n_{\mathrm{select}} = 2^{11}$, $|E^{\mathrm{SCI}}(n_{\mathrm{select}}) - E^{\mathrm{FCI}}|$ drops down to approx. $5 \cdot 10^{-11}$ Ha where it remains for subsequent n_{select}.

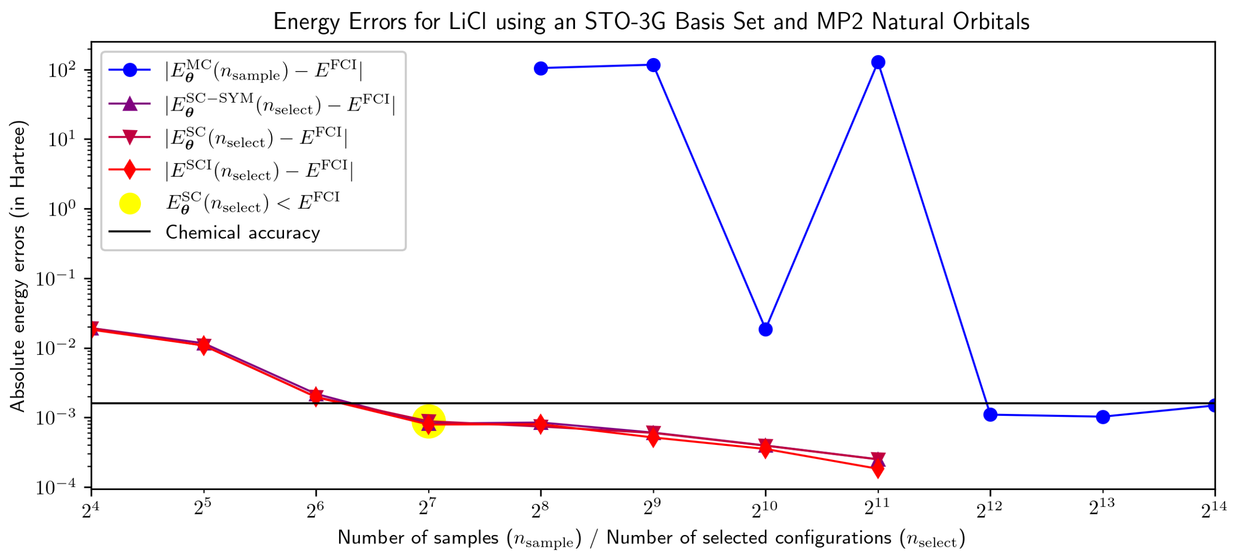

Figure 10: Energy errors for the NBF solutions plotted in Figure 3.

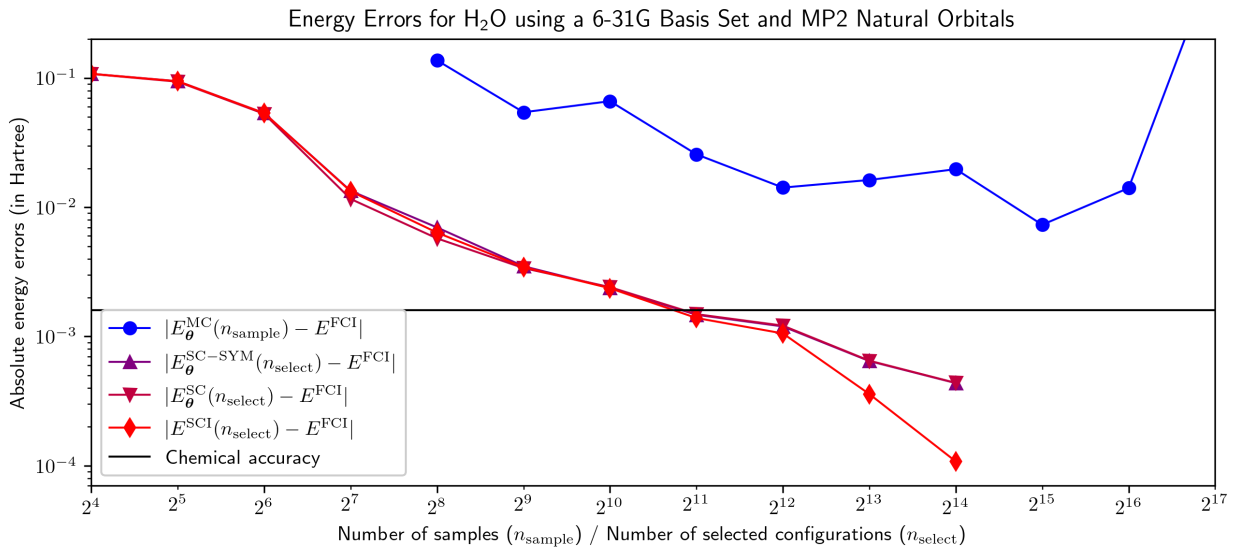

Figure 11: Energy errors for the NBF solutions plotted in Figure 4. $|E_{\boldsymbol{\theta}}^{\mathrm{MC}}(n_{\mathrm{sample}}) - E^{\mathrm{FCI}}|$ for $n_{\mathrm{sample}} = 2^{17}$ is approx. 837 mHa.

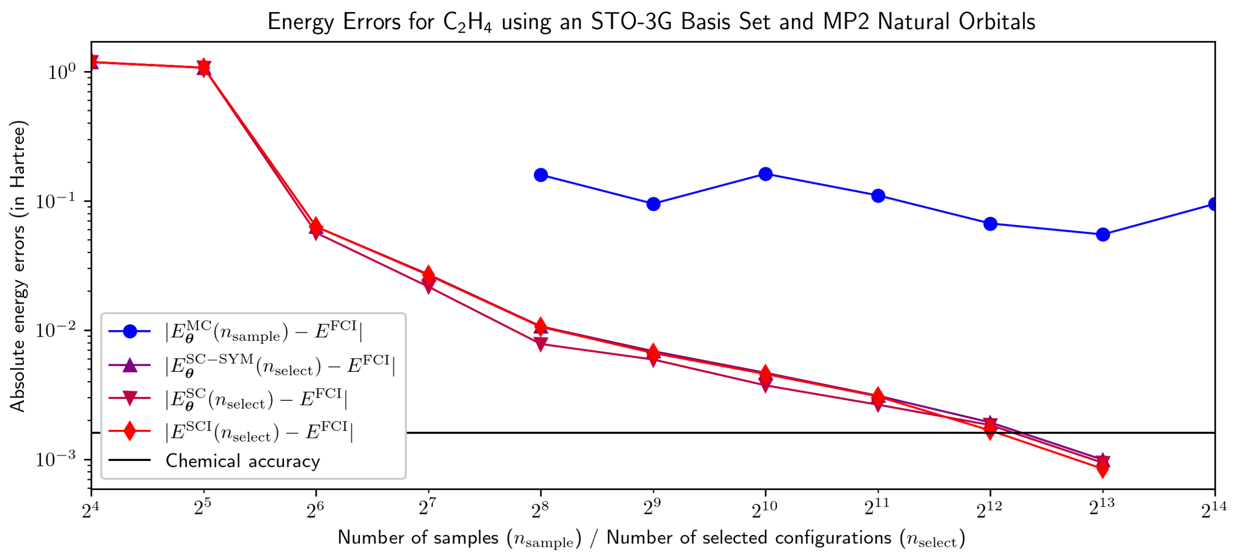

Figure 12: Energy errors for the NBF solutions plotted in Figure 5.

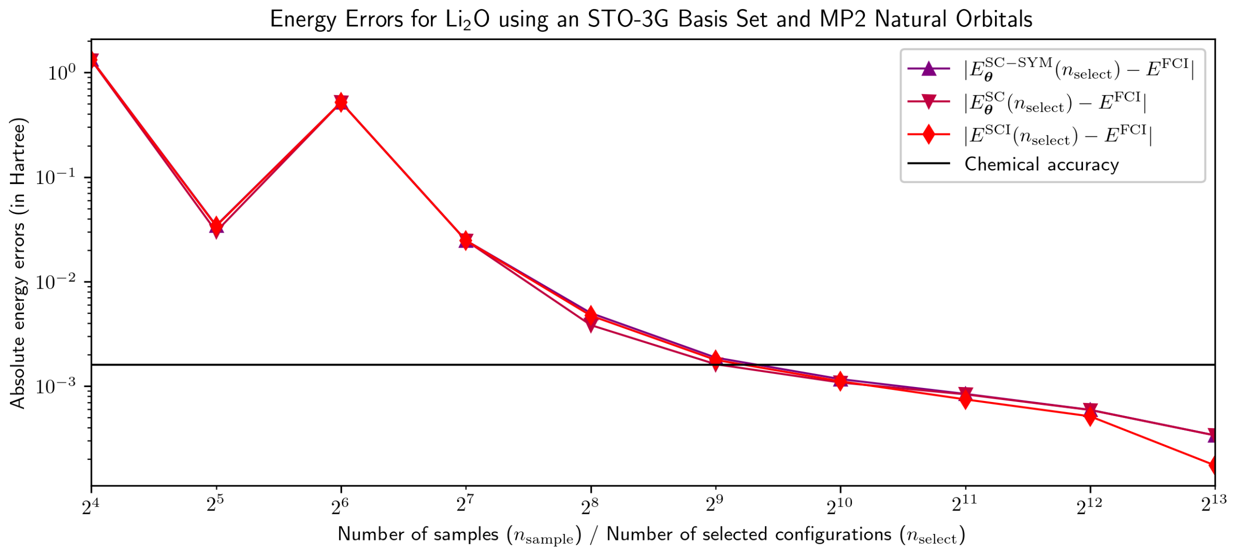

Figure 13: Energy errors for the NBF solutions plotted in Figure 6.

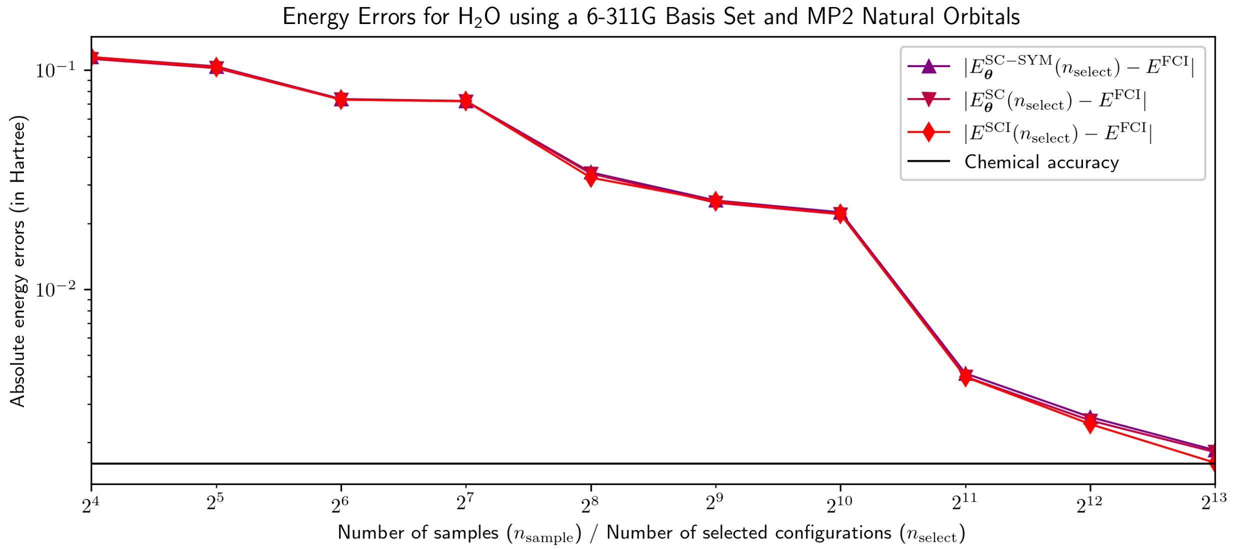

Figure 14: Energy errors for the NBF solutions plotted in Figure 7.

References

(1) Kingma, D. P.; Ba, J. Adam: A Method for Stochastic Optimization. *arXiv:1412.6980* **2017**,

(2) Carleo, G.; Choo, K.; Hofmann, D.; Smith, J. E.; Westerhout, T.; Alet, F.; Davis, E. J.; Efthymiou, S.; Glasser, I.; Lin, S.-H. et al. NetKet: A machine learning toolkit for many-body quantum systems. *SoftwareX* **2019**, *10*, 100311.

(3) Vicentini, F.; Hofmann, D.; Szabó, A.; Wu, D.; Roth, C.; Giuliani, C.; Pescia, G.; Nys, J.; Vargas-Calderón, V.; Astrakhantsev, N. et al. NetKet 3: Machine Learning Toolbox for Many-Body Quantum Systems. *SciPost Phys. Codebases* **2022**, 7.

(4) Bradbury, J.; Frostig, R.; Hawkins, P.; Johnson, M. J.; Leary, C.; Maclaurin, D.; Necula, G.; Paszke, A.; VanderPlas, J.; Wanderman-Milne, S. et al. JAX: Composable Transformations of Python+NumPy Programs. 2018; `http://github.com/jax-ml/jax`, date of access: 2026-02-01.

(5) Heek, J.; Levskaya, A.; Oliver, A.; Ritter, M.; Rondepierre, B.; Steiner, A.; van Zee, M.

Flax: A Neural Network Library and Ecosystem for JAX. 2024; `http://github.com/google/flax`, date of access: 2026-02-01.

(6) Sun, Q. Libcint: An efficient general integral library for Gaussian basis functions. *J. Comput. Chem.* **2015**, *36*, 1664–1671.

(7) Sun, Q.; Berkelbach, T. C.; Blunt, N. S.; Booth, G. H.; Guo, S.; Li, Z.; Liu, J.; McClain, J. D.; Sayfutyarova, E. R.; Sharma, S. et al. PySCF: the Python-based simulations of chemistry framework. *WIREs Comput. Mol. Sci.* **2018**, *8*, e1340.

(8) Sun, Q.; Zhang, X.; Banerjee, S.; Bao, P.; Barbry, M.; Blunt, N. S.; Bogdanov, N. A.; Booth, G. H.; Chen, J.; Cui, Z.-H. et al. Recent developments in the PySCF program package. *J. Chem. Phys.* **2020**, *153*, 024109.

(9) Richer, M.; Sánchez-Díaz, G.; Martínez-González, M.; Chuiko, V.; Kim, T. D.; Tehrani, A.; Wang, S.; Gaikwad, P. B.; de Moura, C. E. V.; Masschelein, C. et al. PyCI: A Python-scriptable library for arbitrary determinant CI. *J. Chem. Phys.* **2024**, *161*, 132502.

(10) NIST Standard Reference Database Number 101, NIST Computational Chemistry Comparison and Benchmark Database. National Institute of Standards and Technology (NIST), 2022; date of access: 2025-12-01.
